# ZO-2 blocks the localization of PGAM5 protein to the mitochondrial membrane and suppresses pancreatic cancer malignancy through metabolic reprogramming

**DOI:** 10.1016/j.gendis.2026.102290

**Published:** 2026-06-24

**Authors:** Sen Yu, Mingxin Ke, Muyuan Ma, Tingting Jiang, Xiaojia Li, Keping Xie

**Affiliations:** a Center for Pancreatic Cancer Research, The South China University of Technology School of Medicine, Guangzhou, Guangdong 510006, China; b The South China University of Technology Comprehensive Cancer Center, Guangzhou, Guangdong 510006, China

**Keywords:** Metabolism, Mitochondria, Pancreas, PGAM5, Tumorigenesis, ZO-2

## Abstract

Pancreatic ductal adenocarcinoma (PDAC) is one of the leading causes of cancer-related death with limited treatment options, and there is an urgent need to develop effective therapeutic strategies. The zonula occludens-2 (ZO-2) protein is a member of the tight junction protein family, and its function and underlying mechanisms of action in PDAC are unknown. Gene expression and its association with the clinicopathologic characteristics of patients with PDAC were analyzed using immunohistochemistry and bioinformatics and functionally validated by using *in vitro* and *in vivo* mouse models. Protein expression and protein regulation were measured by using gain- and loss-of-function assays and molecular biology methods. The expression level of ZO-2 decreased in PDAC and was highly correlated with aggressive pathological features and the prognosis of patients. Increased expression of ZO-2 inhibited the proliferation, migration and colony formation of PDAC cells *in vitro* and inhibited PDAC tumor growth and liver metastasis *in vivo*. Mechanistically, ZO-2 bound to the mitochondrial membrane protein phosphoglycerate mutase 5 (PGAM5) and trapped it in the cytosol. Reduced expression of ZO-2 caused increased mitochondrial localization of PGAM5, leading to activated mitochondrial function and increased intracellular reactive oxygen species and ATP levels, thereby promoting PDAC malignancy, whereas increased ZO-2 expression did the opposite. ZO-2 expression also correlated directly with immune cell infiltration and immune signaling in PDAC. Downregulation of ZO-2 caused increased localization of PGAM5 to the mitochondrial membrane and promoted PDAC malignancy through metabolic reprogramming. ZO-2 is associated with the PDAC immune microenvironment. The ZO-2-PGAM5 axis could serve as a potential therapeutic target in PDAC.

## Introduction

Pancreatic ductal adenocarcinoma (PDAC) is the most important subtype of pancreatic cancer, accounting for more than 90% of cases. In 2020, there were 496,000 new cases and 466,000 deaths from pancreatic cancer worldwide, with an incidence rate almost equal to the mortality rate.^1^ The symptoms of the patients are insidious, and most of them are diagnosed at an advanced stage; 80%–90% of the tumors are unresectable, and 50% of them have metastasis.^2^ At present, the 5-year survival rate of PDAC patients is only 13%,^3^ and the mortality rate is expected to rise to second place among all cancers by 2030.^1^ Therefore, pancreatic cancer is one of the malignant tumors with the worst prognosis, and new treatment methods are urgently needed.

Zonula occludens (ZO) proteins, adaptor proteins of tight junction (TJ) proteins, are located in the proximal cytosol and are responsible for connecting transmembrane TJ proteins with the cytoskeleton to achieve barrier function.^4^ ZO proteins play important roles in various biological processes. Homozygous knockout of ZO-1 or ZO-2 in mice results in embryonic lethality.^5^^,^^6^ The PDZ domain of ZO proteins can bind to oncogenic viruses such as adenovirus and human papillomavirus, leading to the degradation of ZO protein.^7^^,^^8^ Many studies have shown that the development and progression of tumors is frequently accompanied by the loss of ZO proteins, and patients with loss of ZO proteins often have a worse prognosis.^4^ Therefore, ZO proteins may inhibit cancer progression. In PDAC, inhibition of ZO-1 expression promotes the proliferation, migration, invasion and metastasis of tumor cells.^9^ ZO-2 is expressed in PDAC cells and tissues,^10^^,^^11^ but its function in PDAC is unknown.

Phosphoglycerate mutase 5 (PGAM5) is a mitochondrial protein that regulates the activity of binding proteins through its phosphatase function, thereby altering mitochondrial functions, including mitochondrial fission, movement, biogenesis and mitophagy.^12^ PGAM5 can mediate the dephosphorylation of DRP1 at Ser637 and increase the protein activity of DRP1, thereby promoting mitochondrial fission.^13^ Mitochondria are important organelles that regulate cell metabolism and metabolic reprogramming, one of the hallmarks of cancer.^14^ Studies have found that PGAM5 is involved in the development and progression of a variety of cancers and plays a crucial role in the metabolic reprogramming of cancer.^12^ However, there have been no studies on PGAM5 in PDAC, and there is limited progress in mitochondrial research in PDAC. Therefore, this study investigates the effects of ZO-2 and PGAM5 on PDAC development and progression and determines their potential as new therapeutic targets for PDAC treatment and efficacy improvement.

## Materials and methods

### Immunohistochemistry and immunofluorescence

For immunohistochemistry, tissue slides were deparaffinized and rehydrated through an alcohol series followed by antigen retrieval with sodium citrate buffer. Endogenous peroxidase in the tissue was inactivated with 3% hydrogen peroxide (H_2_O_2_) in methanol. Then, these slides were blocked with 10% goat serum (Vivacell, Shanghai, China) for 1 h at room temperature and incubated with primary antibodies (anti-ZO-2, CAT#71-1400, Invitrogen, Carlsbad, California, USA) at 4 °C overnight. Antigen expression levels were then detected by horseradish peroxidase (HRP)-conjugated secondary antibodies and 3,3′-diaminobenzidine (DAB) staining (GeneTech, Shanghai, China). Nuclei were counterstained with hematoxylin (Beyotime, Shanghai, China), and images were taken with a microscope (Wisleap, Changzhou, Jiangsu, China).

For immunofluorescence, tissue slides after antigen retrieval were blocked with 10% goat serum for 1 h at room temperature. Cells on glass coverslips were fixed with 4% paraformaldehyde (Biosharp, Hefei, Anhui, China), permeabilized with 0.2% Triton X-100 (Beyotime, Shanghai, China) and blocked with 1% BSA (Beyotime, Shanghai, China) for 1 h at room temperature. Tissue slides or cells were then incubated with primary antibodies (anti-ZO-2, CAT#71-1400, Invitrogen, Carlsbad, California, USA), anti-CK-19 (CAT#ab52625, Abcam, Cambridge, Cambridgeshire, UK), anti-amylase (CAT#sc-46657, Santa, Dallas, Texas, USA), and anti-PGAM5 (28445-1-AP, Proteintech, Wuhan, Hubei, China) at 4 °C overnight. Antigens were detected with fluorescent dye-conjugated secondary antibodies (Abcam, Cambridge, Cambridgeshire, UK, Invitrogen, Carlsbad, California, USA), and nuclei were counterstained with 4′,6-diamidino-2-phenylindole (DAPI) (Abcam, Cambridge, Cambridgeshire, UK) away from light. Images were taken with a fluorescence microscope (Nikon, Shinagawa Ward, Tokyo, Japan).

### Cell lines and culture conditions

The cell lines used in this study included BxPC-3, CFPAC-1, PK-59 and HEK-293T cells. BxPC-3 and PK-59 cells were cultured in RPMI-1640 medium, CFPAC-1 cells were cultured in Iscove's Modified Dulbecco's Medium (IMDM), and HEK-293T cells were cultured in Dulbecco's Modified Eagle's Medium (DMEM). Complete medium contained 10% fetal bovine serum (FBS) and 1% penicillin-streptomycin antibiotics to prevent bacterial contamination. The human normal pancreatic epithelial ductal cells (HPDE), human PDAC cell lines AsPC-1, BxPC-3, CaPan-2, CFPAC-1, HPAC, HPAF-II, MIA-PaCa-2, PANC-1, PK-59, PL45, SW1990, human embryonic kidney 293 (HEK-293) cell line, and mouse Panc02 were purchased from American Type Culture Collection (ATCC) and were maintained in plastic dishes as adherent monolayers. Among those cell lines, HPDE, HPAC, MIA-PaCa-2, PANC-1, PL45, and HEK-293T cells were maintained in DMEM supplemented with 10% fetal calf serum (FBS, Gibco, Carlsbad, California, USA); PK-59, AsPC-1, BxPC-3, SW1990, and Panc02 cells were maintained in RPMI-1640 medium supplemented with 10% FBS; HPAF-II cells were maintained in Minimum Essential Medium (MEM) supplemented with 10% FBS; and CaPan-2 cells were maintained in McCoy's 5A medium supplemented with 10% FBS. All cell lines were cultured in a humidified incubator at 37 °C with 5% CO_2_. All of the cell lines were obtained between 2020 and 2022, routinely tested for mycoplasma contamination within the last 6 months by using PCR, and used at passage numbers < 15 for this study after reception or thawing in our laboratory. The mutational status sourced from the COSMIC (the Catalog of Somatic Mutations In Cancer) database (https://cancer.sanger.ac.uk/cell_lines) of key driver genes of the mainly utilized cell lines BxPC-3, CFPAC-1, PK-59 and Panc02 is shown in Table S1.

### Western blot analysis

Standard Western blotting was carried out using whole-cell protein lysates and primary and secondary antibodies. Primary antibodies included anti-ZO-2 (Invitrogen, Carlsbad, California, USA, 71–1400), anti-Flag (CAT#14793S, Cell Signaling Technology, Boston, Massachusetts, USA), anti-*β*-actin (CAT#60009-1-Ig, Proteintech, Wuhan, Hubei, China), anti-GAPDH (CAT#60004-1-Ig, Proteintech, Wuhan, Hubei, China), anti-*α*-Tubulin (CAT#60031-1-Ig, Proteintech, Wuhan, Hubei, China), anti-PGAM5 (CAT#28445-1-AP, Proteintech, Wuhan, Hubei, China), anti-COXIV (CAT#11242-1-AP, Proteintech, Wuhan, Hubei, China), and anti-ATP1A1 (CAT#14418-1-AP, Proteintech, Wuhan, Hubei, China). Secondary antibodies included goat anti-mouse IgG (*H*+*L*) (CAT#SA00001-1, Proteintech, Wuhan, Hubei, China), HRP conjugate and goat anti-rabbit IgG (*H*+*L*), HRP conjugate (CAT#SA00001-2, Proteintech, Wuhan, Hubei, China), and HRP-conjugated IgG fraction monoclonal mouse anti-rabbit IgG, light chain specific (CAT#SA00001-7L, Proteintech, Wuhan, Hubei, China). The bands were quantified using ImageJ (version 1.5.1) software.

### Transient gene transfection

Small interfering RNA (siRNA, 100 pmol) or plasmids (1 μg) were transfected into PDAC cells through Lipofectamine 3000 (Invitrogen, Carlsbad, California, USA) for 48 or 72 h before functional assays were performed. siRNA sequences targeting different mRNAs were synthesized by GenePharma (Suzhou, Jiangsu, China) and are listed as follows: ZO-2, 5′-GAACAUGUCUUUAACGGAU-3’; PGAM5, 5′-AAAUCGUCCAUUCGUCUAU-3’. Full-length human ZO-2 or mouse ZO-2 was subcloned and inserted into the control vector plasmid pcDNA3.1 (GenSmart, Nanjing, Jiangsu, China) with a C-terminal Flag tag to generate human pcDNA3.1-ZO-2 (“*pZO-2*”) and mouse pcDNA3.1-ZO-2 (“*pZO-2*”) plasmids.

### Cell proliferation assay

Cells were seeded into 96-well plates at 3 × 10^3^–5 × 10^3^ cells per well and maintained under normal conditions. Cell viability was evaluated by a cell counting kit-8 (CCK-8, Dojindo, Kumamoto, Kumamoto, Japan) assay once a day for 4 days. At each scheduled time point, 10 μL of the CCK-8 reagent and 100 μL of the corresponding cell culture medium were mixed and added to each well. Then, the cells were incubated for 2 h at 37 °C, and the 450 nm absorbance was measured with a microplate reader (Tecan, Männedorf, Switzerland).

### Colony formation assay

Cells were seeded into 6-well plates at 3 × 10^3^–5 × 10^3^ cells per well and maintained under normal conditions for 2–3 weeks. Cells were then fixed with 4% paraformaldehyde and stained with a 0.1% crystal violet solution for 10 min at room temperature. Colonies were counted using ImageJ (version 1.5.1) software.

### Cell scratch wound healing assay

Cells were cultured in 6-well plates until confluence. Wounds were generated by scraping cells with 200-μL tips. Then, the wells were washed with phosphate-buffered saline (PBS) three times and cultured in medium with no serum or 1% serum. Wounds were photographed by microscopy (Leica, Wetzlar, Hessian, Germany) at a magnification of 5 × at 0, 24 and 48 h. Cell migration was assessed by measuring gap areas in multiple fields using ImageJ (version 1.5.1) software.

### Animals

Pathogen-free C57BL/6J mice were purchased from Hunan SJA Laboratory Animal Co., Ltd. (Changsha, Hunan, China). The animal experiments were carried out in strict accordance with the recommendations in the Guide for the Care and Use of Laboratory Animals of the South China University of Technology. The animal protocol was approved by the committee on ethics of animal experiments of the South China University of Technology.

### Animal models of tumor growth and metastasis

For the tumor growth assay, 1 × 10^6^cells in 100 μL PBS were injected subcutaneously into the right dorsal region of nude mice or C57BL/6J mice, or 5 × 10^5^ cells in 50 μL PBS were injected orthotopically into the pancreas of C57BL/6J mice. The tumor-bearing mice were sacrificed when they became moribund, 6–7 weeks for subcutaneous tumor or 2 weeks for orthotopic tumor after inoculation. Then, the tumors were removed, photographed and weighed. Tumor volumes were estimated as (LxW^2^)/2.

For the tumor metastasis assay, 1 × 10^6^cells in 100 μL PBS were injected into the ileocolic veins of C57BL/6J mice. The mice were sacrificed when they became moribund or 3 weeks after inoculation. Their livers were then removed, and surface metastases were counted and photographed after dividing the liver into individual lobes. All livers were fixed, dehydrated, embedded and cut into sections for hematoxylin and eosin (H&E) staining.

### Animal models of acute pancreatitis and pancreatic tumorigenesis

Acute pancreatitis was induced by pancreatic ductal ligation (PDL) for 4 days or by 8-h intraperitoneal injection of 80 μg/kg caerulein (CAE, MCE, Newton, New Jersey, USA) dissolved in saline for 1 day or 2 consecutive days in 8–10-week-old C57BL/6J mice. The mice were sacrificed, and the pancreases were removed at 48 h after the last injection of CAE on day 2.

Pancreatic tumorigenesis was induced by *LSL-Kras*^*G12D*^; *Pdx1-Cre* (KC) mice, which were obtained by crossing *LSL-Kras*^*G12D*^ and *Pdx1-Cre* (C) mice. All pancreases of 25–35-week-old mice were fixed, dehydrated, embedded and cut into sections for H&E staining or immunostaining.

### Reverse transcription and real-time (RT) PCR

Total RNA was extracted with TRIzol reagent (Invitrogen, Carlsbad, California, USA), and cDNA was obtained with the PrimeScript FAST RT reagent Kit with gDNA Eraser (Takara, Kusatsu, Shiga, Japan). The resulting cDNA was then used for RT-PCR analysis with TB Green Premix Ex Taq (Tli RNase H Plus) (Takara, Kusatsu, Shiga, Japan) in a real-time PCR system (Roche, Basel, Switzerland). The results were normalized to the *β*-actin mRNA in each sample. Primer sequences were synthesized by Sangon Biotech and are listed as follows: human ZO-2, 5′-GGGAAGGTCGCTGCTATTGT-3’ (forward) and 5′-CTCTCGCTGTAGCCACTCC-3’ (reverse); human β-actin, 5′-ATTCCAAATATGAGATGCGTTGTT-3’ (forward) and 5′-GTGGACTTGGGAGAGGACTG-3’ (reverse).

### Liquid chromatography-tandem mass spectrometry (LC-MS/MS) analysis

The ZO-2 protein complexes were fractionated by SDS-PAGE, followed by silver staining. The silver-stained gel bands were cut and digested to peptides, which were then separated by high-performance liquid chromatography with a nanoElute system (Bruker, Billerica, Massachusetts, USA). LC-MS/MS analysis was performed on a timsTOF Pro mass spectrometer (Bruker, Billerica, Massachusetts, USA).

### Co-immunoprecipitation (Co-IP) assay

Cells were lysed with Co-IP buffer (20 mM Tris [pH 7.5], 150 mM NaCl, 1% Triton X-100, protease and phosphatase inhibitors). Lysates were centrifuged and cleared by incubation with 50 μL of Protein G agarose beads (Beyotime, Shanghai, China) and 1 μg of normal rabbit IgG (Cell Signaling Technology, Boston, Massachusetts, USA) for 1 h at 4 °C. The pre-cleared supernatant was subjected to IP with primary antibodies (anti-ZO-2, CAT#71–1400, Invitrogen, Carlsbad, California, USA; anti-PGAM5, CAT#28445-1-AP, Proteintech, Wuhan, Hubei, China) at 4 °C overnight. Then, the protein complexes were collected by incubation with 20 μL of Protein G agarose beads for 2 h at 4 °C. Lysates were centrifuged to obtain agarose beads, and the beads were washed with Co-IP buffer four times. The beads were resuspended in 2 × SDS loading buffer and heated for 10 min at 95 °C, and proteins were analyzed by Western blot.

### Mitochondrial and cytosolic protein isolation

Cellular suspensions (≥ 1 × 10^7^ cells) were washed with ice-cold PBS via centrifugation (800 × *g*, 5 min). Lysis in preactivated cytosolic extraction buffer (protease/phosphatase inhibitors, 1 mM DTT) was achieved using 30 ice-cold homogenizer strokes, followed by sequential centrifugation (3000 × *g*, 10 min; 12,000 × *g*, 30 min) to remove nuclear debris. Mitochondrial pellets were resuspended in inhibitor-supplemented buffer, vortexed, and centrifuged (15,000 × *g*, 10 min) to eliminate contaminants. Solubilization utilized ice-cold SDS-Tris lysis buffer (0.5% SDS, 50 mM Tris–HCl, pH 8.0) with intermittent sonication (40 W, 5 s pulses), followed by centrifugation (15,000 × *g*, 10 min). Denatured proteins were aliquoted and stored at −80 °C under liquid nitrogen.

### Membrane protein extraction

Cellular samples (≥ 1 × 10^7^ cells) were washed thrice with ice-cold buffer via centrifugation (3000 rpm, 5 min, 4 °C). Lysates were ice-incubated for 10 min and centrifuged (14,000 rpm, 10 min, 4 °C), and supernatants were subjected to phase separation (37 °C, 10 min; room-temperature centrifugation at 14,000 rpm, 5–20 min) to isolate the lower organic phase. Membrane proteins were purified through two cycles of ice-cold sterile water treatment (4 °C, 5 min) with centrifugation to retain the organic phase, followed by storage at −80 °C. For electrophoretic analysis, 100 μL of extract was acetone-precipitated in an ice bath (20 min), centrifuged (12,000 rpm, 20 min), vacuum-dried, reconstituted in loading buffer and boiled (5 min).

### MitoTracker mitochondrial probe staining

A 200 μM stock solution was prepared by dissolving 50 μg probe powder in 460 μL anhydrous DMSO, aliquoted (10 μL/tube), and stored at −80 °C under vacuum. Working solutions (20–200 nM for live cells; 50–500 nM for fixed samples) were diluted in Ca^2+^/Mg^2+^-supplemented Hanks' balanced salt solution (HBSS) prewarmed at 37 °C for 10 min. Adherent cells were incubated with working solution (37 °C, 20 min, dark), washed thrice with HBSS, and maintained in prewarmed medium. Suspension cells were pelleted (1000 × *g*, 5 min), resuspended in working solution, incubated and washed. Imaging was completed within 30 min; the excitation intensity or probe concentration was reduced if signal attenuation occurred. For fixation, cells were treated with 4% paraformaldehyde (37 °C, 15 min), washed, permeabilized with 0.2% Triton X-100 (10 min, RT), and processed for immunofluorescence.

### Mitochondrial reactive oxygen species (ROS) measurement

A 200 μM working aliquot was prepared by diluting 50 μL stock (1 μg/μL) in 420 μL anhydrous DMSO, aliquoted (10 μL/tube), foil-protected, and stored at −80 °C. For live-cell staining, aliquots were diluted 1:1000–1:10,000 in prewarmed HBSS or medium (20–200 nM final concentration). Adherent cells were incubated in probe-containing medium (37 °C, 5% CO_2_) for 10–30 min, washed thrice with prewarmed medium, and stabilized in fresh medium with antifade reagents. Fluorescence was captured using microscopy (Ex/Em: 579/599 nm).

### Intracellular ROS measurement

The 2′,7′-dichlorodihydrofluorescein diacetate (DCFH-DA) stock solution (≥ 10 mM) was diluted 1:1000 in prechilled HBSS buffer to prepare a 10 μM working solution, with a solvent control prepared in parallel. Target cells (1 × 10^6^–5 × 10^6^ cells/mL) were resuspended in a 3x volume of the working solution and incubated at 37 °C in the dark for 20 min. Then, the cells were washed thrice with prechilled buffer (200 × *g*, 3 min), followed by treatment with test compounds, 50–100 μM H_2_O_2_ (positive control), or solvent (negative control). Spatiotemporal ROS distribution was captured by fluorescence microscopy (Ex/Em = 488/525 nm), and all experiments were completed within 120 min.

### ATP concentration measurement

Adherent cells were lysed with lysis buffer (1/10 culture volume, 4 °C). Lysates were centrifuged (12,000 × *g*, 5 min, 4 °C), and supernatants were collected. A 0.01–10 μM ATP standard curve was generated by serial dilution in lysis buffer. Detection working solution (1:9 reagent/diluent) was ice-thawed. For measurement, 100 μL working solution was equilibrated (room temperature, 3–5 min), followed by sample addition (10–100 μL). Chemiluminescence values were immediately quantified via a multifunctional microplate detector.

### Transmission electron microscopy

Cells were fixed with 2.5% glutaraldehyde, postfixed with 1% osmium tetroxide, dehydrated with ethanol and acetone, and embedded in epoxy resin. Seventy-nanometer sections were cut using a Leica EM UC7 microtome (Leica). Ultrathin sections were negatively stained with uranyl acetate and lead citrate. Images were acquired using a Talos L120C G2 electron microscope equipped with a 4k × 4k Ceta CMOS camera (Thermo Fisher Scientific).

### Statistical analysis

GraphPad Prism (version 10.1.2, Boston, Massachusetts, USA) software was used to perform statistical analysis. Data are shown as the mean ± standard deviation (SD). Categorical data were analyzed using the χ^2^ test or Fisher's exact test, and numerical data from independent experiments were analyzed using the two-tailed Student's *t* test or one-way analysis of variance (ANOVA). All experimental data were examined in at least three independent experiments. *P* values less than 0.05 were considered statistically significant. ∗*p* < 0.05, ∗∗*p* < 0.01, ∗∗∗*p* < 0.001, ∗∗∗∗*p* < 0.0001, ns, not significant.

## Results

### ZO-2 was down-regulated in PDAC and associated with poor prognosis

We used the Cancer Genome Atlas (TCGA) public PDAC dataset to compare the expression levels of ZO-2 in human normal pancreatic tissues and pancreatic tumor tissues, based on which PCA, cluster analysis and differential expression analysis were performed. The results showed that there was an obvious separation between ZO-2 expression levels in pancreatic cancer tissues and normal tissues (Fig. S1A–S1D), and ZO-2 expression was significantly decreased in tumor tissues (Fig. S1C and S1D). In addition, the overall survival of patients with low ZO-2 expression was significantly shorter than that of patients with high ZO-2 expression (Fig. S1B). We performed association analysis between ZO-2 and cancer invasive phenotype-related proteins and found that ZO-2 was positively correlated with the expression level of E-cadherin but negatively correlated with the expression level of N-cadherin (Fig. S1E), indicating that ZO-2 may affect PDAC invasion.

**Figure 1:**
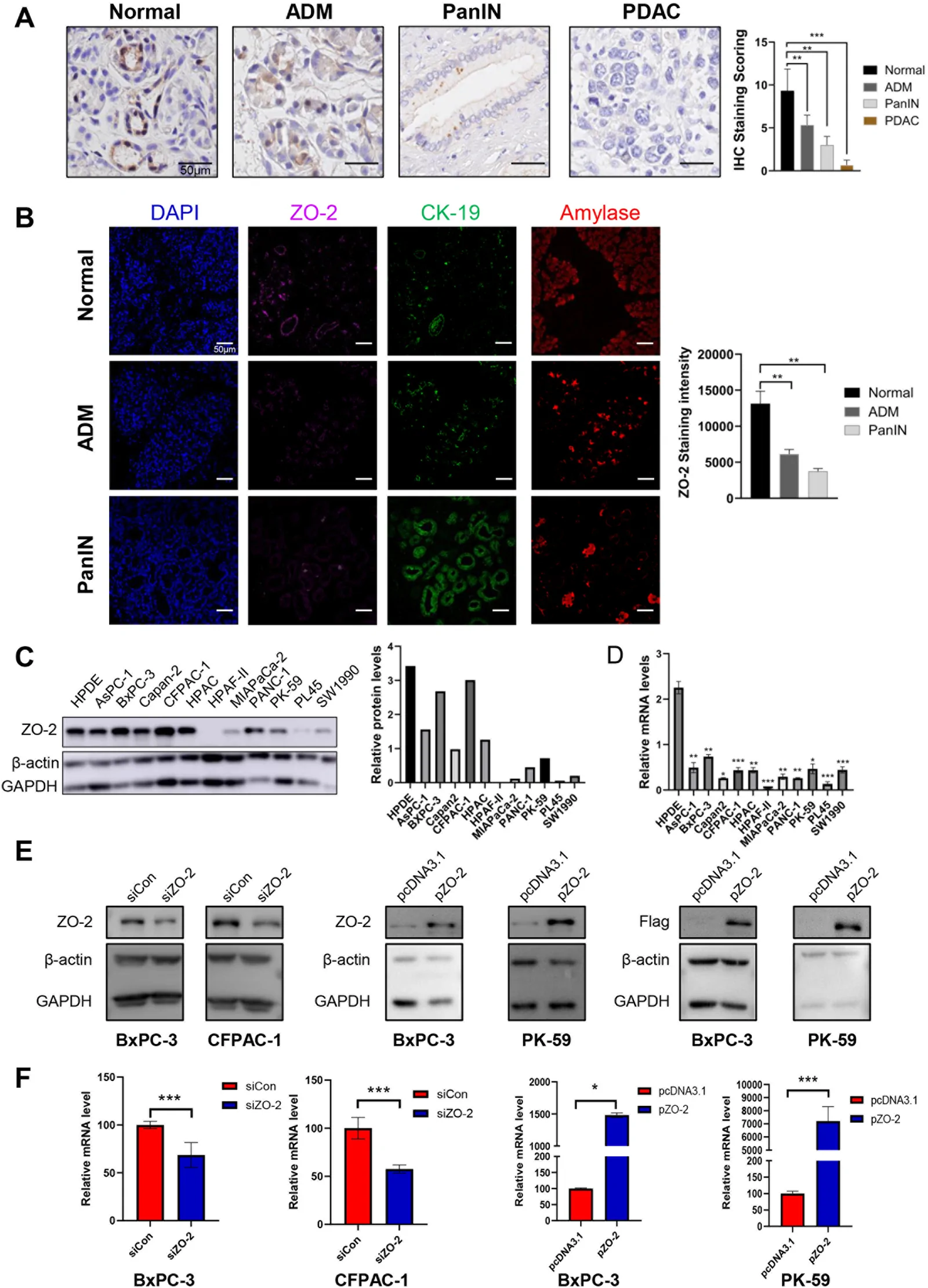
ZO-2 was downregulated in pancreatic cancer. **(A)** Immunohistochemical staining of ZO-2 in PDAC tissues and adjacent normal pancreatic tissues (left, 80 × magnification). Statistical analysis of the ZO-2 staining intensity (right, *n* = 3). **(B)** Fluorescence staining of ZO-2 in PDAC tissues and adjacent normal pancreatic tissues (20 × magnification, *n* = 3). A CK-19 antibody was used to mark ductal cells, and an amylase antibody was used to mark acinar cells. Nuclei were stained with DAPI. The ZO-2 staining intensity was calculated with ImageJ software. **(C)** Western blot analysis of the expression levels of ZO-2 protein in the normal HPDE and PDAC cell lines. **(D)** qPCR analysis of the expression levels of ZO-2 mRNA in HPDE and PDAC cell lines. **(E)** Western blot analysis of the expression levels of ZO-2 protein in PDAC cell lines BxPC-3, CFPAC-1 and PK-59 transfected with siCon, siZO-2, pcDNA3.1 or pZO-2 carrying Flag tag protein. **(F)** qPCR analysis of the expression levels of ZO-2 mRNA in BxPC-3, CFPAC-1 and PK-59 PDAC cell lines transfected with siCon, siZO-2, pcDNA3.1 or pZO-2 carrying Flag tag protein.

To further determine the expression and distribution of ZO-2 in PDAC tissues and cells, we performed immunohistochemistry (Fig. 1A) and immunofluorescence staining on normal pancreas, acinar-to-ductal metaplasia, pancreatic intraepithelial neoplasias and PDAC (labeling acinar cells with an anti-amylase antibody and ductal cells with an anti-CK-19 antibody, Fig. 1B). There was a significant loss of ZO-2 in early lesions and cancer tissues relative to the adjacent normal pancreas. ZO-2 was mainly co-localized with CK-19, indicating that ZO-2 was mainly expressed in ductal cells (Fig. 1B). Consistently, single-cell PDAC data analysis confirmed that ZO-2 was expressed primarily in ductal cells (Fig. S2A). The protein and mRNA expression levels of ZO-2 were significantly decreased in PDAC cell lines relative to HPDE (Fig. 1C and D). These results suggest that reduced ZO-2 may be involved in PDAC pathogenesis.

To determine the tumor biological functions of ZO-2 in PDAC, BxPC-3, CFPAC-1 and PK-59 cell lines with moderate expression levels of ZO-2 protein were selected for subsequent experiments. ZO-2 knockdown was achieved by transfection of siRNA using BxPC-3 and CFPAC-1 cells, while ZO-2 overexpression was achieved by transfection of ZO-2 expression plasmid carrying Flag tag using BxPC-3 and PK-59 cells. Western blotting and RT-PCR were used to examine the expression of ZO-2 at the protein level (Fig. 1E) and RNA level (Fig. 1F), respectively, and the expression levels of ZO-2 were significantly changed.

### ZO-2 inhibited pancreatic cancer progression *in vitro* and *in vivo*

**Figure 2:**
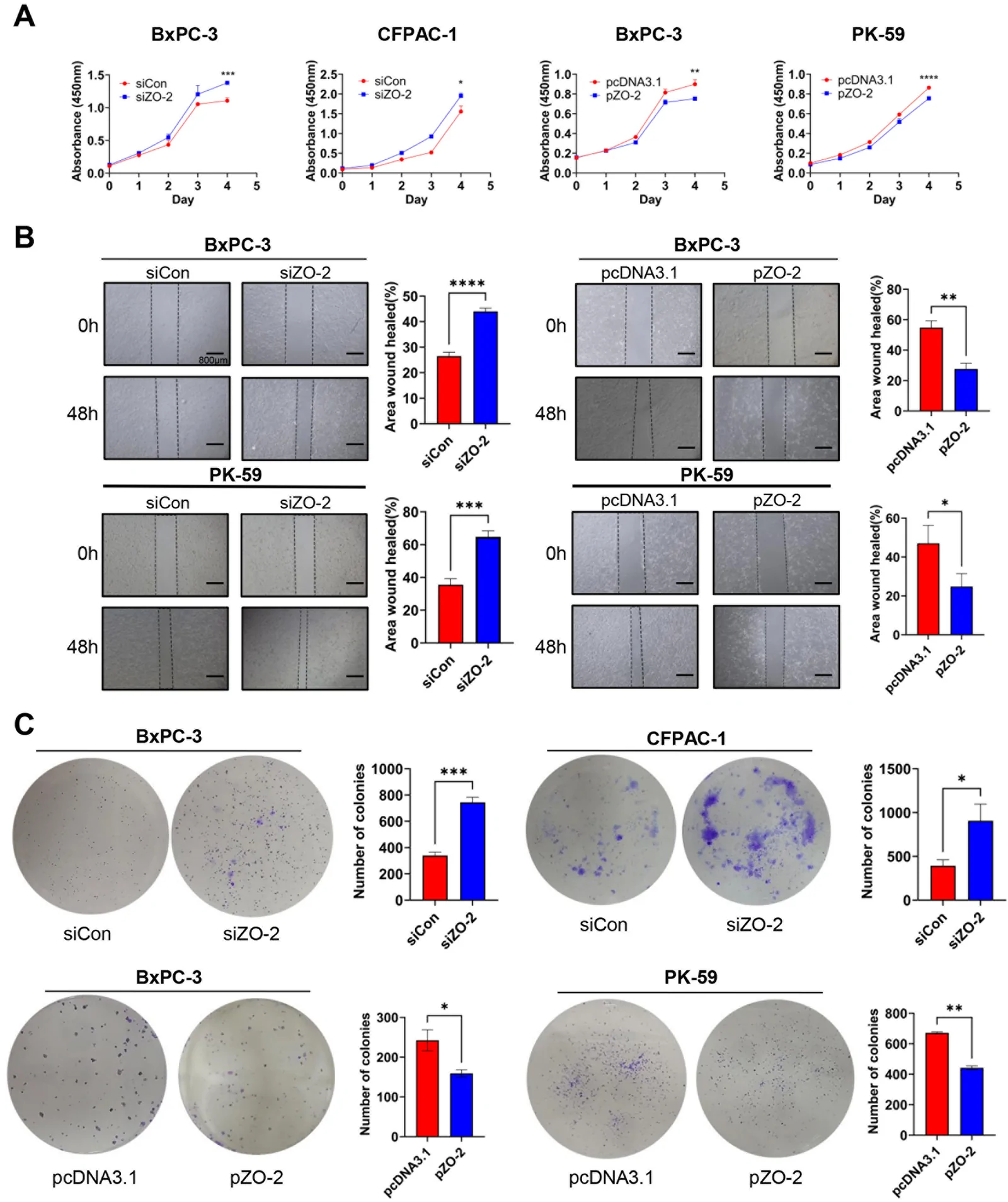
ZO-2 inhibited human pancreatic cancer malignancy *in vitro*. **(A)** The viability of BxPC-3, CFPAC-1 and PK-59 cells transfected with siCon, siZO-2, pcDNA3.1 or pZO-2 was evaluated by CCK-8 assay (*n* = 3). **(B)** The wound areas of BxPC-3 and PK-59 cells transfected with siCon, siZO-2, pcDNA3.1 or pZO-2 were measured with ImageJ software after scraping cells with 200-μL tips (5 × magnification, *n* = 3). **(C)** Colonies formed by BxPC-3, CFPAC-1 and PK-59 cells transfected with siCon, siZO-2, pcDNA3.1 or pZO-2 were fixed with paraformaldehyde and stained with crystal violet, and colony numbers were determined with ImageJ software (*n* = 3).

To investigate the effect of ZO-2 on the proliferation ability of human PDAC cells, we examined the proliferation ability of PDAC cells from 0 to 4 days by CCK-8 assay. The proliferation ability of BxPC-3 and CFPAC-1 cells significantly increased after ZO-2 knockdown (Fig. 2A), while the proliferation ability of BxPC-3 and PK-59 cells was significantly weakened after ZO-2 overexpression (Fig. 2A), indicating that ZO-2 inhibited the proliferation of human PDAC cells. To investigate the effect of ZO-2 on the migratory ability of PDAC cells, we performed a scratch assay. ZO-2 knockdown significantly accelerated wound healing in BxPC-3 and CFPAC-1 cells and enhanced cell migration ability (Fig. 2B), whereas ZO-2 overexpression reduced cell migration ability in BxPC-3 and PK-59 cells (Fig. 2B), indicating that ZO-2 inhibited the migration of human PDAC cells. In a colony formation assay, ZO-2 knockdown significantly increased the number and sizes of colonies in BxPC-3 and CFPAC-1 cells, while ZO-2 overexpression significantly reduced the number and sizes of colonies in BxPC-3 and PK-59 cells (Fig. 2C), indicating that ZO-2 inhibited colony formation in human PDAC cells.

**Figure 3:**
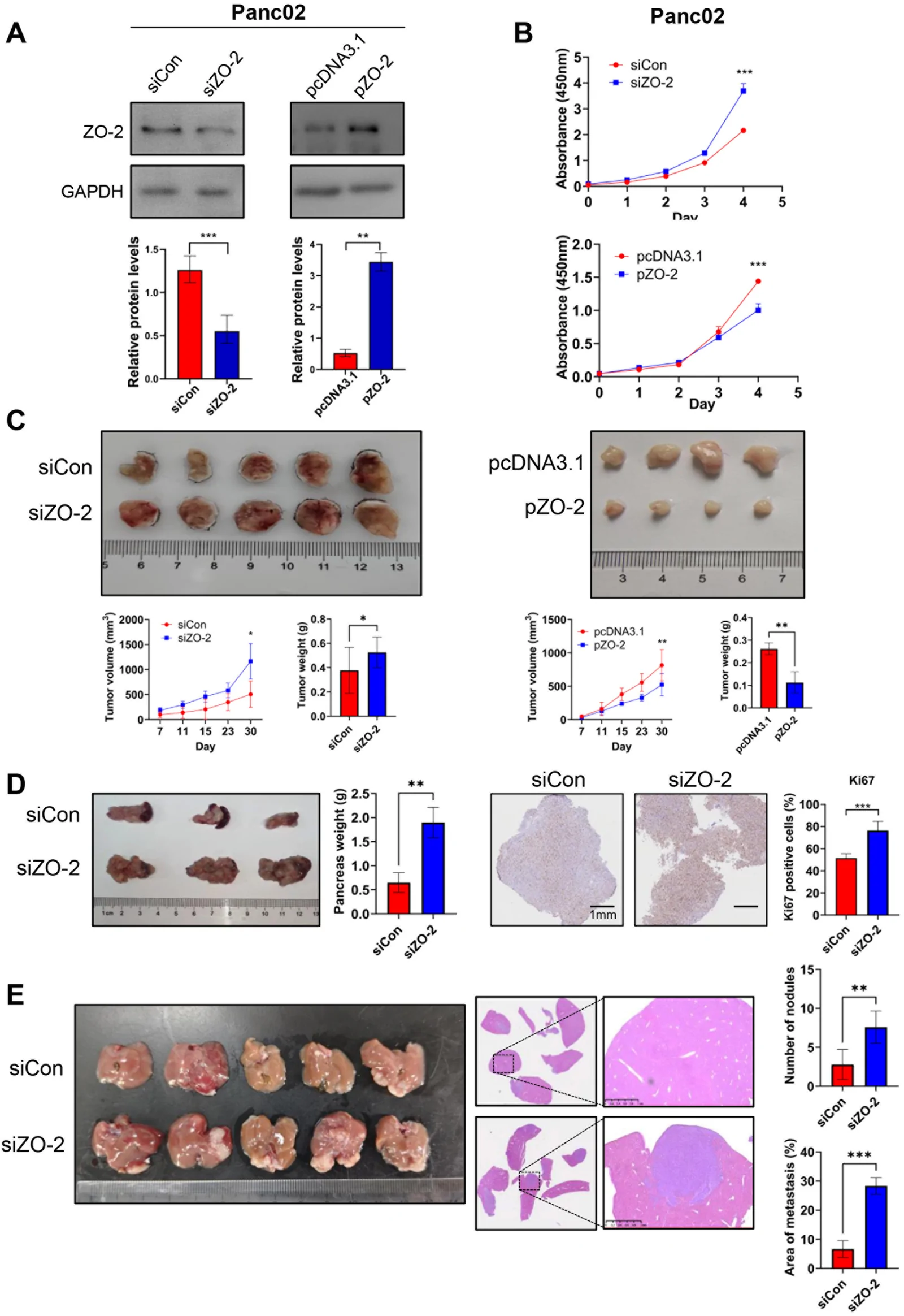
ZO-2 inhibited mouse pancreatic cancer malignancy *in vivo*. **(A)** Western blot analysis of the expression levels of ZO-2 protein in Panc02 PDAC cells transfected with siCon, siZO-2, pcDNA3.1 or pZO-2 carrying a Flag tag. **(B)** The viability of Panc02 cells transfected with siCon, siZO-2, pcDNA3.1 or pZO-2 was evaluated by the CCK-8 assay (*n* = 3). **(C)** Subcutaneous tumor volumes of Panc02 cells transfected with siCon, siZO-2, pcDNA3.1 or pZO-2 were measured once a week. The tumors were removed and weighed one month after tumor cell injection (*n* = 4, 5). **(D)** Orthotopic tumors of Panc02 cells transfected with siCon or siZO-2 were removed and weighed 2 weeks after tumor cell injection. The tissue slides were stained with a Ki67 antibody (*n* = 3×, 10 × magnification). **(E)** The livers of a mouse liver metastasis model of Panc02 cells transfected with siCon or siZO-2 for 2 weeks were collected. Hematoxylin and eosin staining was performed on the tissue slides. The number of nodules was counted, and the area of metastasis was measured with ImageJ software (*n* = 5×, left, 0.5 × magnification, right, 5 × magnification).

To determine the function of ZO-2 in mouse PDAC cells, we first performed ZO-2 knockdown and overexpression in Panc02 mouse PDAC cells using siRNA and an expression plasmid, respectively (Fig. 3A). Next, we used the CCK-8 assay, scratch assay and colony formation assay to detect the effect of ZO-2 interference on the biological function of Panc02 cells. Consistent with the results of ZO-2 in human PDAC cells, ZO-2 knockdown significantly increased the proliferation ability (Fig. 3B), migration ability and colony formation ability (Fig. S2B and S2C) of Panc02 cells, whereas ZO-2 overexpression significantly suppressed Panc02 cell proliferation (Fig. 3B), migration and colony formation (Fig. S2B and S2C).

To determine the effect of ZO-2 on the tumor growth of PDAC cells *in vivo*, a subcutaneous mouse model and an orthotopic pancreatic model were used. PDAC cells with ZO-2 knockdown produced larger tumors with higher proliferation ability in subcutaneous and orthotopic tumors, whereas overexpression of ZO-2 did the opposite (Fig. 3C and D; Fig. S3A and S3B). In addition, ZO-2 knockdown induced more severe liver metastasis (Fig. 3E). Therefore, ZO-2 is critical for PDAC progression.

### ZO-2 bound to the mitochondrial membrane protein PGAM5 and was associated with mitochondrial function

**Figure 4:**
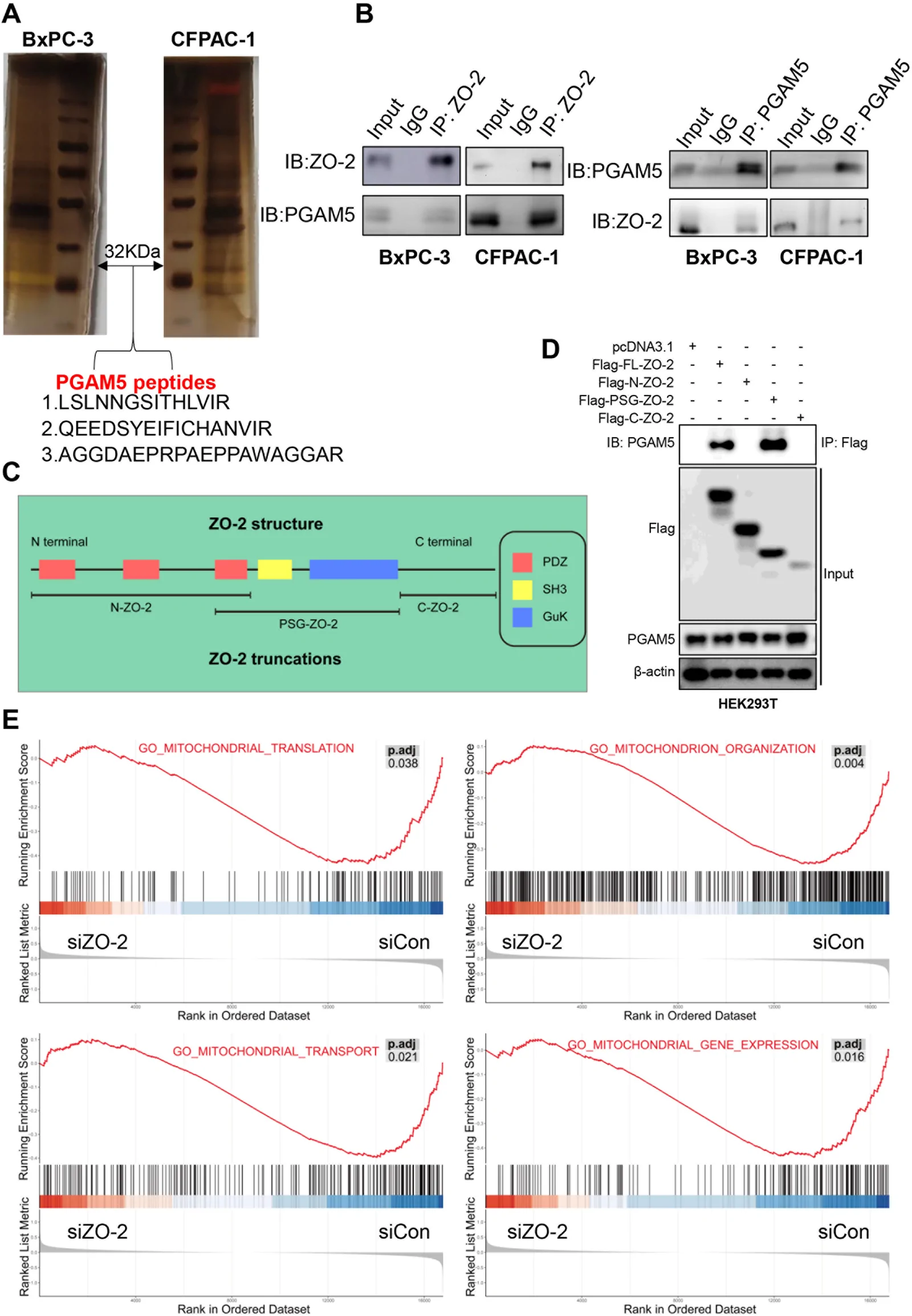
ZO-2 bound to the mitochondrial membrane protein PGAM5 and was associated with mitochondrial function. **(A)** ZO-2 protein complexes from BxPC-3 and CFPAC-1 cells were fractionated by SDS-PAGE, followed by silver staining. The silver-stained gel bands were cut and digested to peptides, which were then subjected to liquid chromatography-tandem mass spectrometry (LC-MS/MS) analysis. **(B)** Protein lysates from BxPC-3 and CFPAC-1 cells were subjected to co-immunoprecipitation (Co-IP) with a ZO-2 antibody or a PGAM5 antibody, and ZO-2 and PGAM5 expression was then determined by Western blot analysis. **(C)** Schematic diagram of the ZO-2 structure and its truncations. **(D)** Protein lysates from HEK-293T cells transfected with pcDNA3.1 or ZO-2 truncations (Flag-FL-ZO-2, Flag-N-ZO-2, Flag-PSG-ZO-2, Flag-C-ZO-2) for 48 h were subjected to Co-IP with a Flag antibody, and then Western blot analysis of PGAM5 and Flag was carried out with corresponding antibodies. β-actin served as a loading control. **(E)** Gene set enrichment analysis (GSEA) of BxPC-3 and CFPAC-1 cells with or without ZO-2 knockdown (*n* = 3).

Given the function of ZO-2 in communicating cell signals by binding to other signaling proteins, we used immunoprecipitation and liquid chromatography-tandem mass spectrometry (LC-MS/MS) to screen the interacting proteins of ZO-2 and explore the mechanism of ZO-2 function in PDAC. Peptide sequence analysis showed that ZO-2 bound to multiple peptides of the mitochondrial membrane protein PGAM5 (Fig. 4A), and secondary mass spectra are shown (Fig. S4A and S4B), suggesting that PGAM5 is a potential binding protein. Co-IP experiments of ZO-2 and PGAM5 were performed in BxPC-3 and CFPAC-1 cells, and ZO-2 and PGAM5 bound to each other (Fig. 4B).

To explore which domain of ZO-2 binds to PGAM5, we first constructed three truncated ZO-2 fragments carrying Flag-tagged proteins based on the distribution characteristics of key domains (PDZs, SH3, GuK) of ZO-2 (Fig. 4C): the N-ZO-2 fragment located at the N-terminus, containing three PDZ domains; the PSG-ZO-2 fragment located in the middle, containing one PDZ domain, one SH3 domain and one GuK domain; and the C-ZO-2 fragment located at the C-terminus, not containing any known key domains. Subsequently, we overexpressed the three ZO-2 truncated fragments (Flag-N-ZO-2, Flag-PSG-ZO-2, Flag-C-ZO-2) as well as the negative control pcDNA3.1 or full-length ZO-2 (Flag-FL-ZO-2) in HEK-293T cells for 48 h and then performed Co-IP experiments. The results showed that only the Flag-FL-ZO-2 protein and the Flag-PSG-ZO-2 fragment bound PGAM5 protein (Fig. 4D), indicating that it is the PSG domain of ZO-2 that binds to PGAM5.

To test whether ZO-2 affected mitochondrial function, we first performed RNA-Seq after ZO-2 knockdown in BxPC-3 and CFPAC-1 cells. Clustering analysis indicated that gene expression patterns changed after ZO-2 knockdown in both cell lines (Fig. S5A), and the differentially expressed genes are shown with volcano plots (Fig. S5B). Gene set enrichment analysis (GSEA) showed that the expression level of ZO-2 significantly influenced mitochondrial signaling, such as mitochondrial translation, organization, transport and gene expression (Fig. 4E). We performed GSEA on a public GEO PDAC dataset (GSE62452) containing 69 PDAC tissues according to ZO-2 expression levels, and similar roles of ZO-2 in mitochondrial signaling were observed (Fig. S6). These results suggested that ZO-2 may affect mitochondrial function through PGAM5 and thereby affect PDAC cell biological function.

### ZO-2 inhibited mitochondrial function by impeding the mitochondrial localization of PGAM5

**Figure 5:**
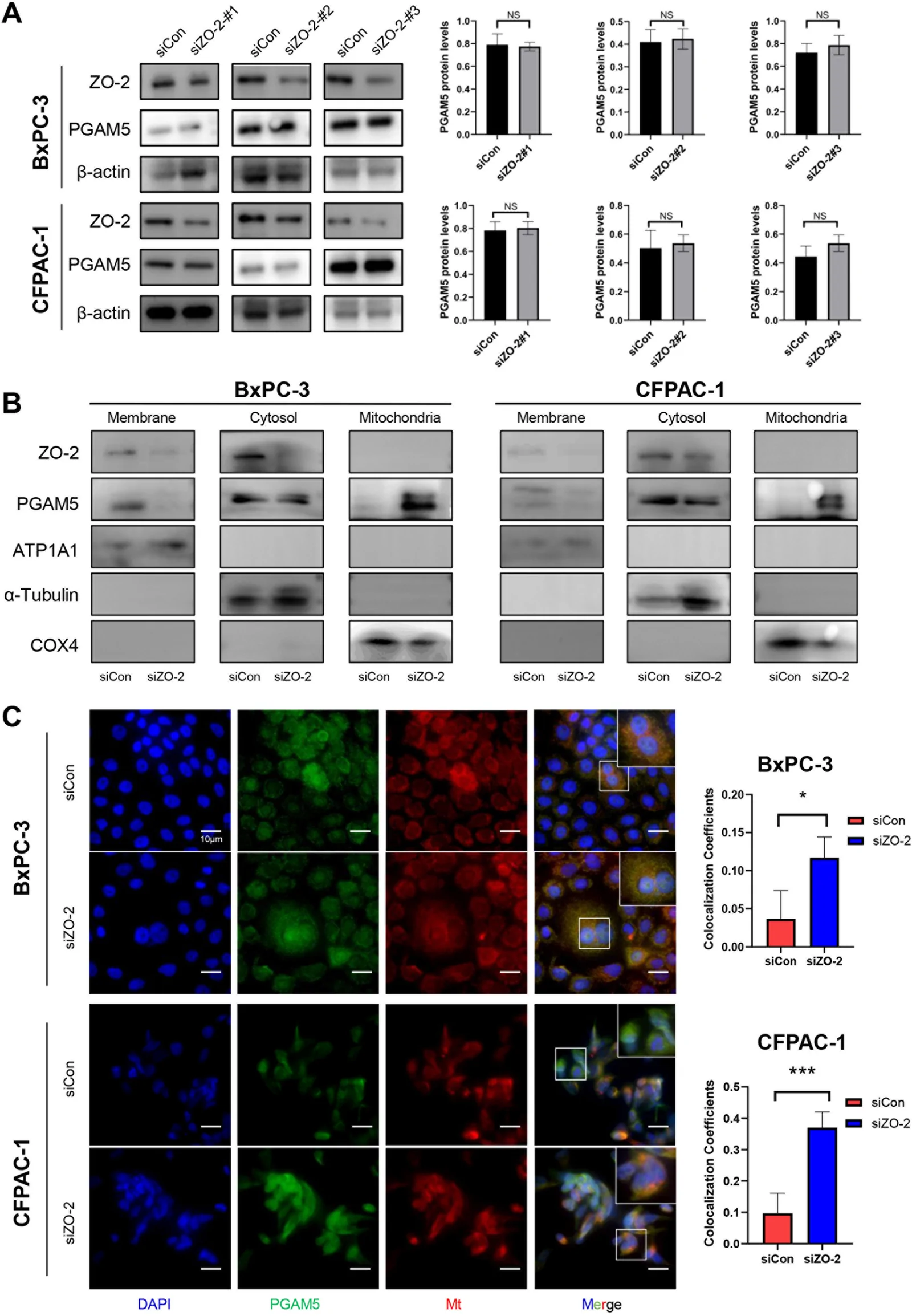
ZO-2 impeded PGAM5 mitochondrial localization. **(A)** Three independent Western blot analyses of the expression levels of ZO-2 and PGAM5 proteins in BxPC-3 and CFPAC-1 PDAC cells transfected with siCon or siZO-2. **(B)** Western blot analysis of the protein expression levels of ZO-2 and PGAM5 in the cell membrane, cytosol and mitochondria in PDAC BxPC-3 and CFPAC-1 cells transfected with siCon or siZO-2. ATP1A1, α-Tubulin and COX4 served as loading controls for the cell membrane, cytosol and mitochondria, respectively. **(C)** Fluorescence staining of PGAM5 and mitochondria with a PGAM5 antibody and MitoTracker Red CMXRos fluorescent probe, respectively, in BxPC-3 and CFPAC-1 cells transfected with siCon or siZO-2 (40 × and 100 × magnification, *n* = 3). Nuclei were stained with DAPI, and colocalization coefficients were calculated with ImageJ software.

To further examine the biological significance of the interaction between ZO-2 and PGAM5, we first examined the protein content of PGAM5 in ZO-2 knockdown PDAC cell lines. Knockdown of ZO-2 in PDAC cell lines did not significantly affect the total protein expression level of PGAM5 (Fig. 5A). Then, mitochondrial, cytosolic and membrane proteins were extracted before and after ZO-2 knockdown, and the expression levels of PGAM5 were detected. ZO-2 knockdown significantly increased the expression level of PGAM5 in mitochondria, while the expression level of PGAM5 around the cell membrane and in the cytosol was significantly decreased (Fig. 5B). Considering that ZO-2 is a juxtamembrane cytosolic protein, the results suggested that ZO-2 could trap PGAM5 in the juxtamembrane cytosol and hinder its translocation to mitochondria, although ZO-2 does not affect the expression level of PGAM5. To further confirm this notion, MitoTracker Red CMXRos fluorescent probe was used to label mitochondria with simultaneous PGAM5 immunofluorescence staining. The proportion of PGAM5 colocalized with mitochondria was significantly increased in ZO-2 knockdown PDAC cells compared to control PDAC cells (Fig. 5C), indicating that ZO-2 inhibited the transport of PGAM5 to mitochondria. Meanwhile, we employed transmission electron microscopy (TEM) to examine the effects of ZO-2 on mitochondrial morphology. The results revealed that the proportion of cells exhibiting mitochondrial fission significantly increased following ZO-2 knockdown (Fig. S7).

**Figure 6:**
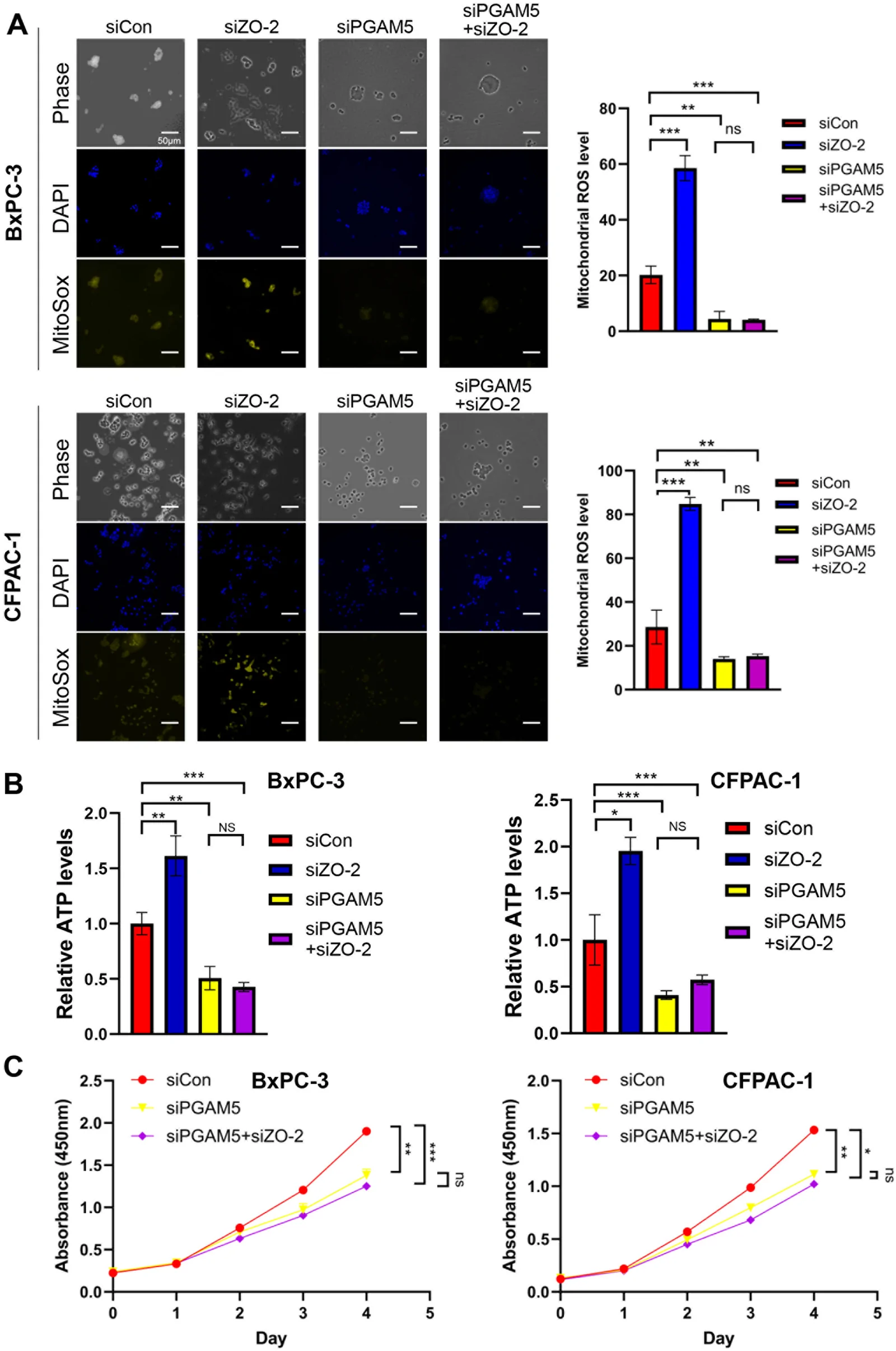
ZO-2 impaired mitochondrial functions through PGAM5. **(A)** Mitochondrial ROS levels were detected with the MitoSOX fluorescent probe and ImageJ software in BxPC-3 and CFPAC-1 PDAC cells transfected with siCon, siZO-2, siPGAM5 or siPGAM5+siZO-2 (20 × magnification, *n* = 3). **(B)** ATP levels were detected with luciferase and fluorescein in BxPC-3 and CFPAC-1 cells transfected with siCon, siZO-2, siPGAM5 or siPGAM5+siZO-2. **(C)** The viability of BxPC-3 and CFPAC-1 cells transfected with siCon, siPGAM5 or siPGAM5+siZO-2 was evaluated by the CCK-8 assay (*n* = 3).

To test whether ZO-2 affected mitochondrial function, mitochondrial reactive oxygen species (ROS) levels were measured using a MitoSOX fluorescent probe, intracellular ROS levels were measured using a DCFH-DA fluorescent probe, and intracellular ATP levels were measured using luciferase and fluorescein. We found that mitochondrial ROS levels were significantly increased in PDAC cells with ZO-2 knockdown (Fig. 6A), and intracellular ROS levels (Fig. S8B) and ATP levels (Fig. 6B) were also significantly increased. In contrast, ZO-2 overexpression significantly decreased mitochondrial and intracellular ROS levels (Fig. S8A and S8B).

To determine the effect of PGAM5 on mitochondrial function, we also measured the levels of ROS and ATP after PGAM5 knockdown and found that the levels of mitochondrial ROS (Fig. 6A), intracellular ROS (Fig. S8B) and ATP (Fig. 6B) decreased significantly after PGAM5 knockdown. Further experiments showed that after knockdown of both ZO-2 and PGAM5 at the same time, the levels of ROS and ATP were still significantly down-regulated (Fig. 6A and B; Fig. S8B), indicating that PGAM5 mediated the effect of ZO-2 on mitochondrial function.

To determine the effect of PGAM5 on PDAC cell proliferation, CCK-8 assays were performed after PGAM5 knockdown or knockdown of both ZO-2 and PGAM5. Either PGAM5 knockdown alone or combined knockdown of ZO-2 and PGAM5 significantly decreased cell proliferation (Fig. 6C), indicating that PGAM5 was required for the effect of ZO-2 on PDAC cell proliferation. To further investigate the relationship between ZO-2 and PGAM5, we performed rescue experiments. The results demonstrated that while PGAM5 knockdown significantly inhibited cell colony formation (Fig. S9B), migration (Fig. S9A), growth (Fig. S10B), and ATP production (Fig. S10A), the overexpression of ZO-2 failed to rescue or alter these phenotypes. In summary, ZO-2 inhibited mitochondrial function by impeding PGAM5 mitochondrial localization, thereby affecting PDAC development and progression (Fig. 9).

**Figure 7:**
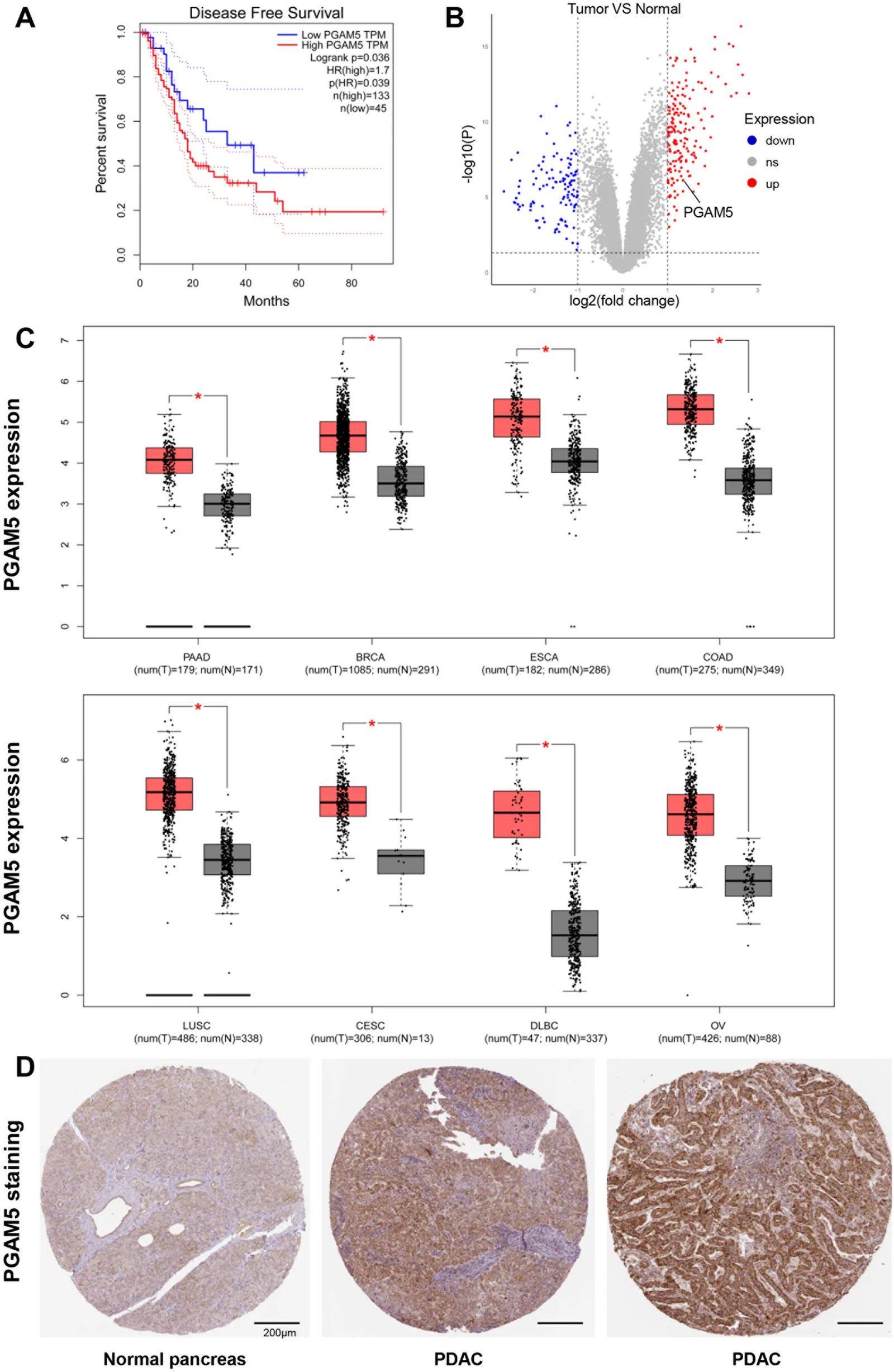
PGAM5 was up-regulated in pancreatic cancer and was associated with poor prognosis in the TCGA and Human Protein Atlas datasets. **(A)** Survival analysis of PDAC patients with high and low PGAM5 expression levels. **(B, C)** Differential expression analysis of PDAC tissues, other cancer tissues and normal tissues. **(D)** Immunohistochemical staining of PGAM5 in PDAC tissues and normal pancreas tissues.

Moreover, we used the TCGA and Human Protein Atlas public datasets to compare the expression levels of PGAM5 in human normal tissues and tumor tissues, based on which survival analysis and differential expression analysis were performed. The results showed that the disease-free survival of patients with high PGAM5 expression was significantly shorter than that of patients with low expression (Fig. 7A). In addition, there was an obvious separation between PGAM5 expression levels in various cancer tissues and normal tissues, and PGAM5 expression was significantly increased in tumor tissues, including PDAC tissues (Fig. 7B–D), indicating that PGAM5 may be a potential PDAC therapeutic target.

### ZO-2 was associated with changes in the tumor microenvironment

**Figure 8:**
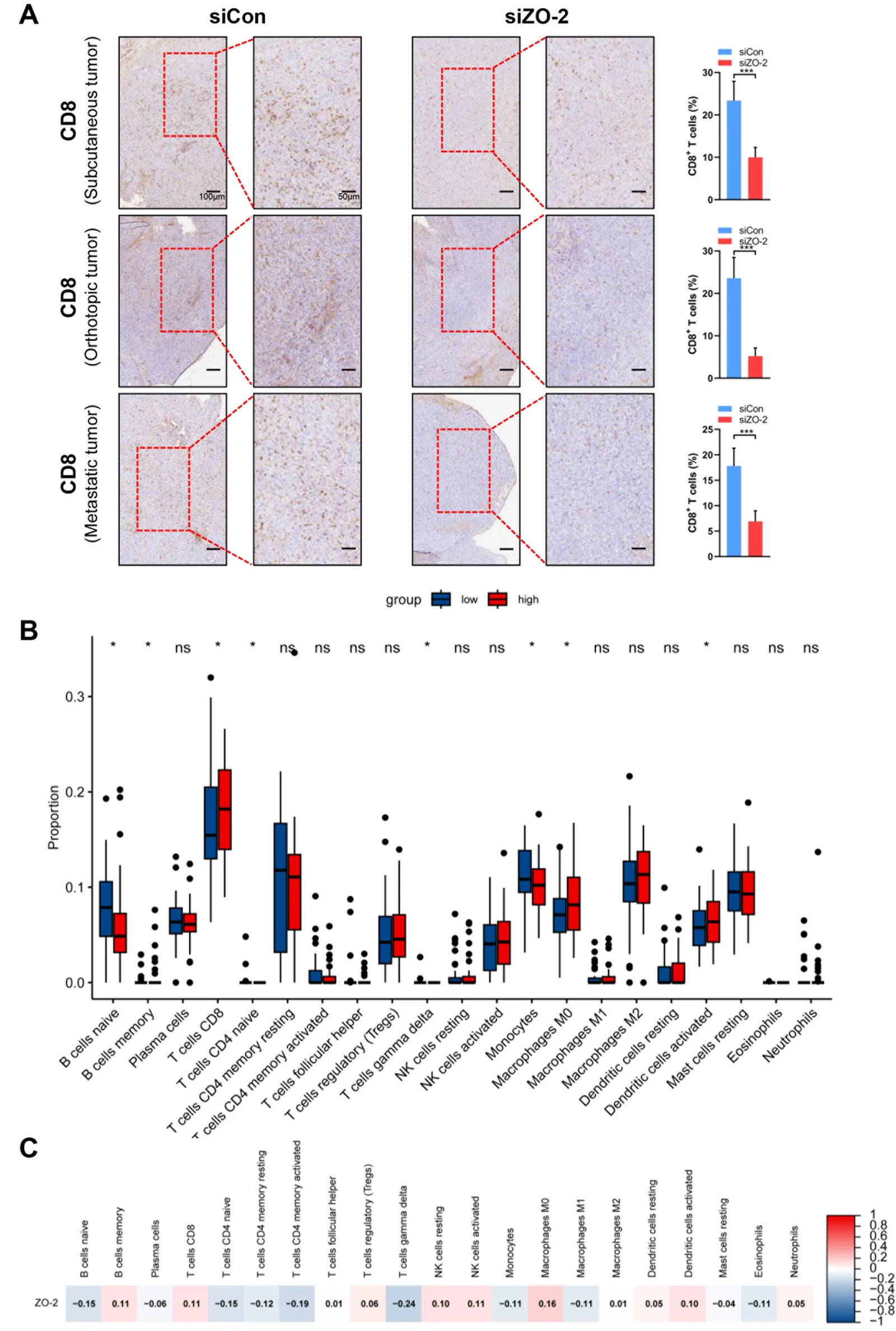
Correlation of altered ZO-2 expression with changes in infiltrating immune cells in the tumor microenvironment. **(A)** Immunohistochemical staining of CD8 in subcutaneous, orthotopic and metastatic tumors (10 × magnification, *n* = 5). **(B)** Analysis of various types of immune cell infiltration using the public PDAC dataset (GSE62452) according to ZO-2 expression levels. **(C)** Analysis of the correlation between various infiltrating immune cells and ZO-2 expression levels using a public PDAC dataset (GSE62452).

Given that dysregulated metabolic reprogramming of tumor cells can influence the tumor microenvironment, we determined the infiltration of CD8^+^ T cells in subcutaneous, orthotopic and metastatic tumors formed by PDAC cells with or without ZO-2 knockdown and found that ZO-2 expression was positively associated with CD8^+^ T cell infiltration (Fig. 8A). Furthermore, we found that ZO-2 was negatively related to T_reg_ cell infiltration with FOXP3 staining (Fig. S11A) and was positively related to several immune signaling pathways, such as interferon, tumor necrosis factor (TNF) and transforming growth factor-beta (TGF-β) (Fig. S11B). Additionally, we performed analyses of various immune cell proportions using a public PDAC dataset (GSE62452) according to ZO-2 expression levels. We found that ZO-2 was associated with immune cell infiltration in tumors, including B cells, CD8^+^ T cells, CD4^+^ T cells, macrophages and dendritic cells, and increased ZO-2 expression was consistently associated with increased CD8^+^ T cell infiltration (Fig. 8B and C; Fig. S12A). Therefore, ZO-2 may also serve as a potential predictive molecule of immune cell infiltration of the tumor microenvironment.

**Figure 9:**
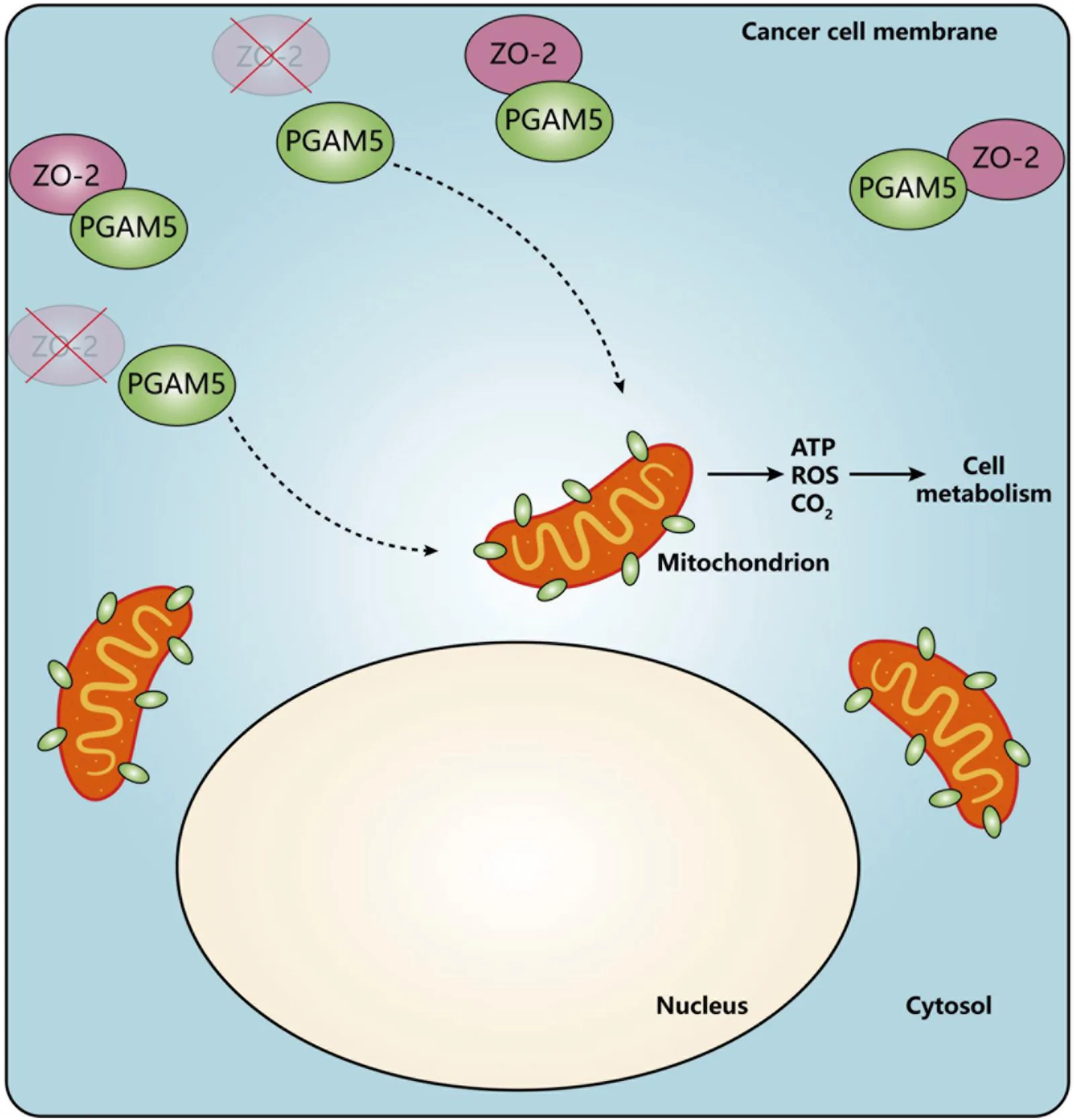
Schematic diagram of the role of the ZO-2-PGAM5 axis in regulating PDAC cell metabolism. Downregulation of ZO-2 causes increased localization of PGAM5 to the mitochondrial membrane and promotes PDAC malignancy through metabolic reprogramming.

To explore how ZO-2 may influence the tumor microenvironment, we performed weighted gene coexpression network analysis (WGCNA) of the public PDAC dataset (GSE62452) to screen for modules and genes related to ZO-2. As a result, the gene set was divided into two modules by average linkage hierarchical clustering (Fig. S12B–S12D). The turquoise module suggested a positive correlation between tumor proportion and ZO-2 and a negative correlation between stromal proportion and ZO-2 (Fig. S12D). Then, the gene set of the turquoise module was subjected to protein–protein interaction (PPI) enrichment analysis and demonstrated that proteins participating in regulating cell secretion or apical cell junctions were associated with changes in the tumor microenvironment (Fig. S13A and S13B).

## Discussion

In the present study, we found that the expression level of ZO-2 was down-regulated in PDAC. ZO-2 knockdown promoted the proliferation, migration and colony formation of PDAC cells *in vitro* and promoted pancreatic tumor growth and liver metastasis *in vivo*. Mechanistically, ZO-2 bound to the mitochondrial membrane protein PGAM5 and trapped it in the cytosol, inhibiting its mitochondrial localization and leading to impaired mitochondrial function and dysregulated cancer cell metabolism. Therefore, ZO-2 blocks the localization of the PGAM5 protein to the mitochondrial membrane and suppresses PDAC malignancy through metabolic reprogramming; therefore, the ZO-2-PGAM5 axis can serve as a potential therapeutic target.

Previous studies of ZO-2 in pathological statuses have focused on familial genetic diseases. For example, the ZO-2 gene mutation could induce familial intrahepatic cholestasis, familial hypercholanemia and nonsyndromic hearing loss.^15^ In addition, ZO-2 plays a critical role in the blood-testis barrier, and ZO-2 knockout causes a permeable blood-testis barrier, smaller testes, reduced fertility and degeneration of seminiferous tubules.^16^ Recently, the tumor suppressor role of ZO-2 in cancer progression has been revealed in non-small cell lung cancer cells; ZO-2 interacts with p190A and p120 RasGAP to activate the Hippo signaling pathway, therefore inhibiting cell proliferation and tumorigenesis.^17^ Moreover, ZNF582 could increase the ZO-2 protein level, which bound to ERK2 to suppress its phosphorylation, therefore inhibiting cell growth and metastasis of clear cell renal cell carcinoma.^18^ In our study, we found that ZO-2 suppressed PDAC development and progression, representing the first discovery of the role of ZO-2 in PDAC.

PGAM5 is a phosphatase located in the mitochondrial inner membrane and can be translocated to the mitochondrial outer membrane or cytosol to perform functions after cleavage by the protease presenilin-associated rhomboid-like.^12^ In our Western blot results, two bands of PGAM5 could be observed, indicating its cleavage, with the upper band as full-length PGAM5 and the lower band as cleaved PGAM5. PGAM5 can dephosphorylate DRP1, the mitochondrial fission GTPase, to activate its activity on the mitochondrial outer membrane^13^ or mitochondrial-associated membrane with the help of the protein STX17,^19^ subsequently promoting mitochondrial fission. KRAS mutation in more than 90% of PDACs persistently activates the MAPK signaling pathway.^20^ The protein kinase ERK2 of the MAPK signaling pathway phosphorylates DRP1 on Serine 616 to promote mitochondrial fission and tumor growth.^21^ Mechanistically, DRP1 impaired glycolytic flux and mitochondrial metabolic function,^22^ and inhibition of DRP1 induced mitochondrial fusion and suppressed PDAC tumor growth.^23^ In addition, PGAM5 could form a protein complex with the transcription factor NRF2 and its key negative regulator KEAP1 to prevent apoptosis induced by excessive ROS levels by enhancing the anti-oxidant gene expression regulated by NRF2.^24^ It is known that KEAP1-NRF2 signaling is critical for regulating PDAC tumor growth, metastasis and therapeutic resistance by influencing oxidative stress.^25^ While the downstream mechanisms of PGAM5 remain to be validated in our current work, we believe these findings provide a valuable basis for future scientific hypotheses.

No study has reported the function of PGAM5 in PDAC, both of which are closely related to mitochondrial function. PGAM5 has been implicated in the regulation of other cancers. For example, PGAM5 was overexpressed in hepatocellular carcinoma and positively correlated with poor prognosis.^26^ Mechanistically, PGAM5 promoted tumor growth and treatment tolerance by binding to the apoptosis-related protein BCL-XL to promote its protein stability, thereby reducing apoptosis.^26^ Moreover, aberrant release of mitochondrial ROS could induce PGAM5 release from mitochondria to the cytosol to dephosphorylate MST3 kinase, which prevents LATS1/2 phosphorylation, leading to YAP activation and accelerated colorectal cancer progression.^27^ Therefore, PGAM5 is closely associated with cancer progression, including PDAC.

In our study, we found for the first time that ZO-2 suppressed PDAC progression through PGAM5. Mechanistically, ZO-2 trapped PGAM5 in the cytosol near the cell membrane, inhibiting its mitochondrial localization and leading to impaired mitochondrial function. Knockdown of ZO-2 promoted PGAM5 translocation from the cytosol to mitochondria. Our finding was consistent with a prior study, which showed that ZO-2 binds to three transcription factors (Jun, Fos and C/EBP) and traps them in the cytosol near the membrane.^28^ Overexpression of ZO-2 significantly down-regulated the expression of genes regulated by these transcription factors. ZO-1 also inhibited the nuclear translocation of the transcription factor ZONAB by binding to and retaining ZONAB in the cytosol near the membrane.^29^ As ZONAB could bind to the cell cycle regulatory proteins CDK4 and cyclin D1, overexpression of ZO-1 also inhibited their nuclear translocation, thereby inhibiting cell proliferation. Consistently, we found that PGAM5 knockdown could impair mitochondrial function and cell proliferation in PDAC, while combined knockdown of PGAM5 and ZO-2 could not reverse the impact, suggesting that PGAM5 is necessary for ZO-2 to exert functions in PDAC.

In summary, ZO-2 blocks the localization of the PGAM5 protein to the mitochondrial membrane and suppresses PDAC malignancy through metabolic reprogramming. In addition, ZO-2 is associated with the PDAC immune microenvironment. Therefore, the ZO-2-PGAM5 axis can serve as a potential therapeutic target. Further in-depth investigation into the interaction and function of ZO-2 and PGAM5 proteins in PDAC would help validate novel molecular targets for PDAC treatment.

## CRediT authorship contribution statement

**Sen Yu:** Conceptualization, Data curation, Formal analysis, Investigation, Methodology, Writing – original draft, Writing – review & editing. **Mingxin Ke:** Formal analysis, Investigation. **Muyuan Ma:** Data curation, Formal analysis. **Tingting Jiang:** Formal analysis, Funding acquisition, Project administration, Writing – review & editing. **Xiaojia Li:** Formal analysis, Investigation, Project administration, Supervision, Writing – review & editing. **Keping Xie:** Writing – review & editing, Writing – original draft, Visualization, Supervision, Software, Resources, Project administration, Methodology, Investigation, Funding acquisition, Formal analysis, Conceptualization.

## Data availability

All available on request.

## Funding

The work is partly supported by the 10.13039/501100001809National Natural Science Foundation of China (No. 82072632) and Guangzhou Municipality Bureau of Science and Technology, Guangzhou, China (No. 202102010033).

## Conflict of interests

Keping Xie is a member of *Genes & Diseases* Editorial Board. To minimize bias, he/she was excluded from all editorial decision-making related to the acceptance of this article for publication. The remaining authors declare no conflict of interests.
