# Supplementary material for: ZO-2 blocks the localization of PGAM5 protein to the mitochondrial membrane and suppresses pancreatic cancer malignancy through metabolic reprogramming
Source: Genes Dis. 2026 Jun 24;14(1):102290. doi: 10.1016/j.gendis.2026.102290 (PMC13589071; doi:10.1016/j.gendis.2026.102290)
Supplement: Supplementary file 1 [file mmc1.pdf]

ZO-2 Blocks Localization of PGAM5 Protein to Mitochondrial Membrane and Suppresses Pancreatic Cancer Malignancy through Metabolic Reprogramming

Sen Yu[1,2#], Mingxin Ke[1,2#], Muyuan Ma[1,2#], Tingting Jiang[1,2], Xiaojia Li[1,2✉], and Keping Xie[1,2✉]

[1]Center for Pancreatic Cancer Research, The South China University of Technology School of Medicine, Guangzhou 510006, China

[2]The South China University of Technology Comprehensive Cancer Center, Guangdong 510006, China

✉*Correspondence*: Dr. Keping Xie or Dr. Xiaojia Li, Center for Pancreatic Cancer Research, South China University of Technology School of Medicine, Guangzhou 510006; Phone: 020-3938-0579; E-mail: mcxiekeping@scut.edu.cn or xiaojia_0424@163.com

[#]S.Y., M.K., and M.M. contribute equally to this work and are considered co-first authors. The work represents partial fulfillment of their PhD/MS/BS requirements and integral parts of their doctoral/master/bachelor theses.

Supplementary Figures

Figure S1

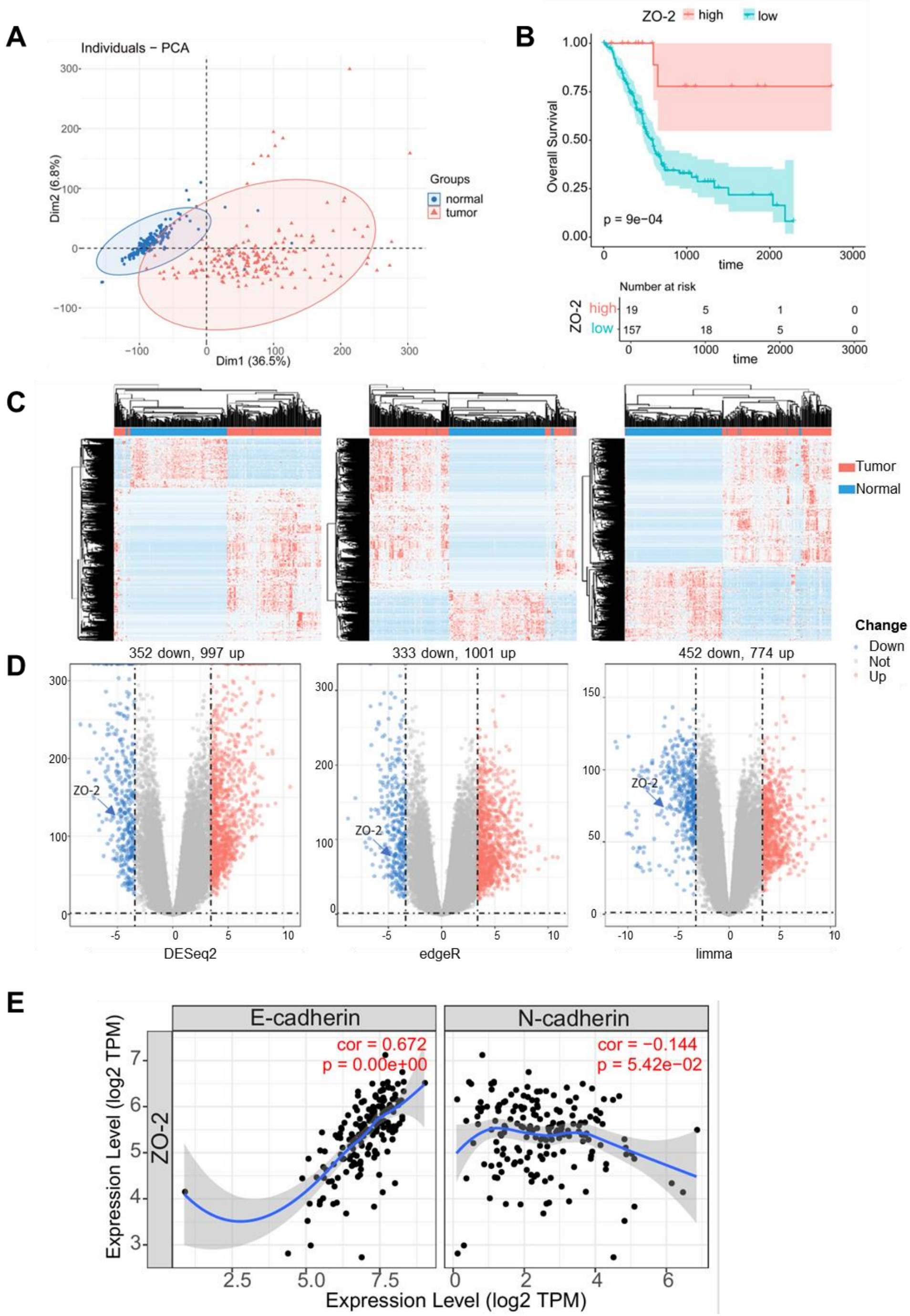

Fig. S1. ZO-2 was down-regulated in pancreatic cancer and was associated with poor prognosis in the TCGA PDAC dataset.

(**A**) PCA analysis of PDAC tissues and normal pancreas tissues. (**B**) Survival analysis of PDAC patients with high and low ZO-2 expression levels. (**C**) Cluster analysis of PDAC tissues and normal pancreas tissues. (**D**) Differential expression analysis of PDAC tissues and normal pancreas tissues. (**E**) Association analysis between the expression levels of ZO-2 and cancer invasive phenotypic-related proteins E-cadherin and N-cadherin in PDAC tissues.

Figure S2

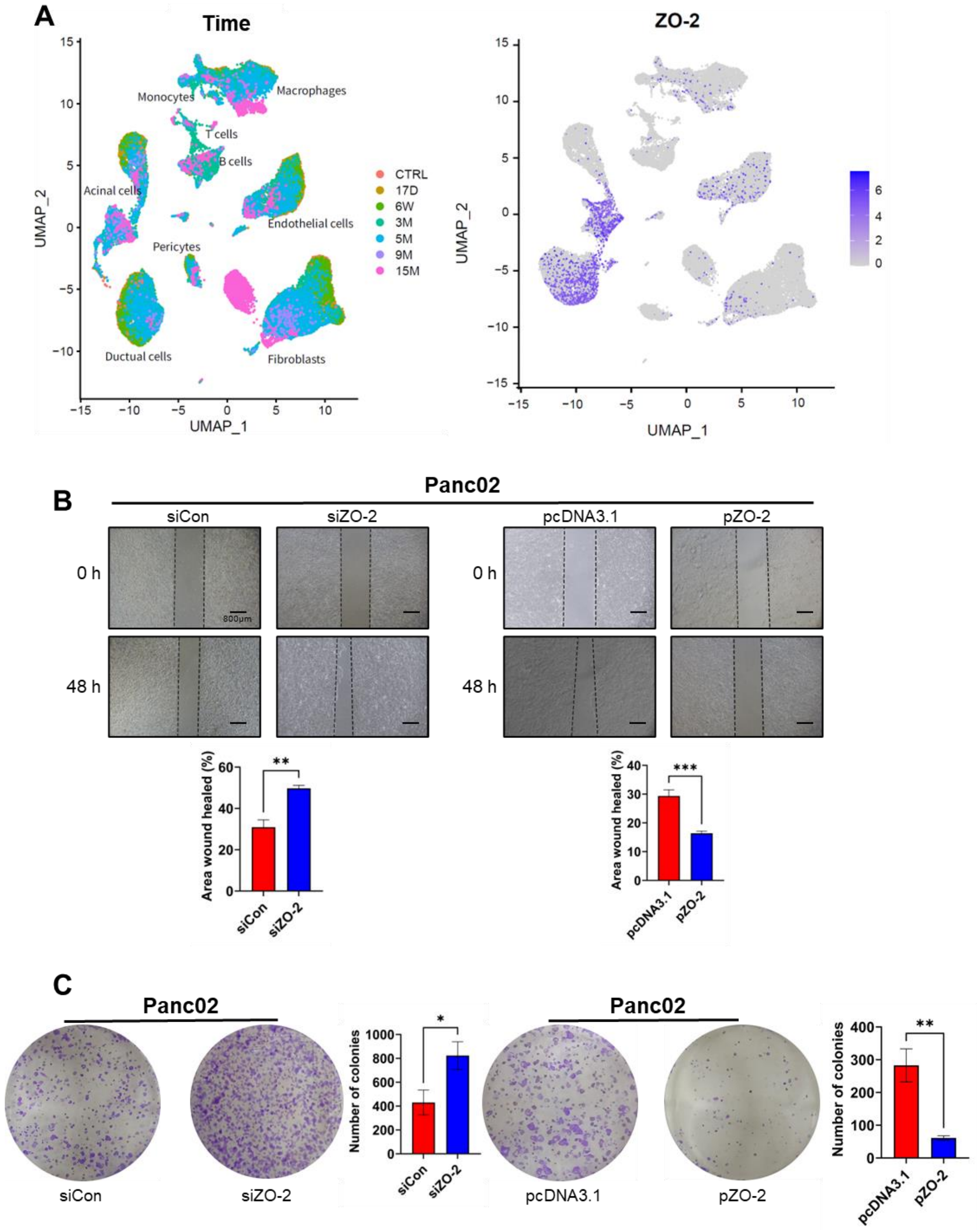

Fig. S2. The ZO-2 mRNA expression patterns in mouse pancreatic precancerous lesions and PDAC, and the role of ZO-2 in Panc02 mouse PDAC cell line.

(**A**) Mouse pancreatic precancerous lesions and PDAC were induced by $Kras^{G12D}$ for 17 days, 6 weeks, 3 months, 5 months, 9 months and 15 months in a single-cell RNA sequencing GEO dataset (GSE141017). (**B**) The wound areas of Panc02 cells transfected with siCon, siZO-2, pcDNA3.1 or pZO-2 were measured with the image J software after scraping cells with 200-μL tips (n=3, 5× magnification). (**C**) Colonies formed by Panc02 cells transfected with siCon, siZO-2, pcDNA3.1 or pZO-2 were fixed with paraformaldehyde and stained with crystal violet, and colony numbers were measured with the image J software (n=3).

Figure S3

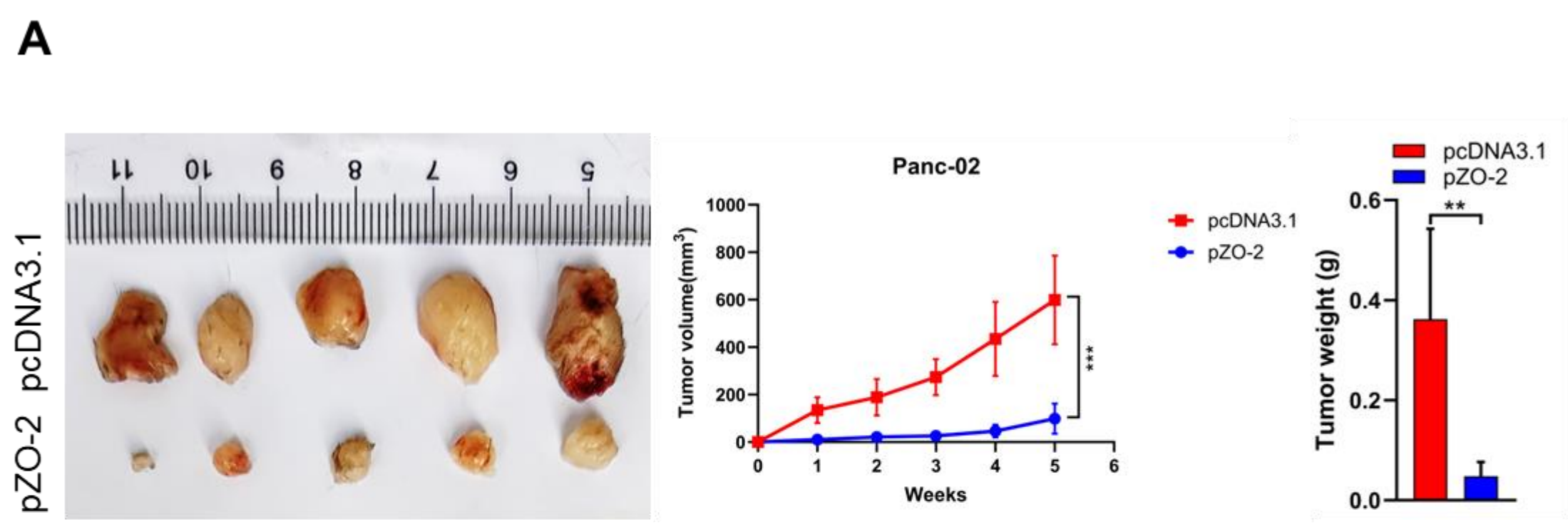

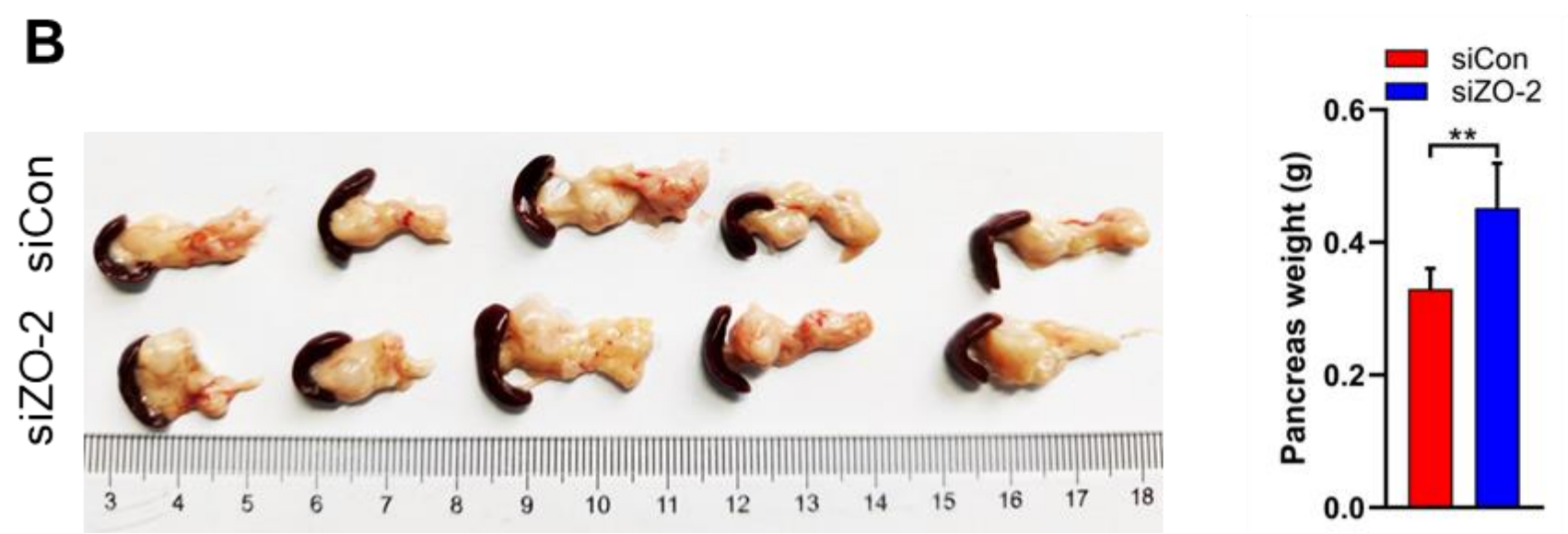

Fig. S3. ZO-2 inhibited mouse pancreatic cancer malignancy *in vivo*.

(A) Subcutaneous tumor volumes of Panc02 cells transfected with pcDNA3.1 or pZO-2 were measured once a week. The tumors were removed and weighed a month after tumor cell injection (n=5). (B) Orthotopic tumors of Panc02 cells transfected with siCon or siZO-2 were removed and weighed 2 weeks after tumor cell injection (n=5).

A

The secondary mass spectrum of ZO-2

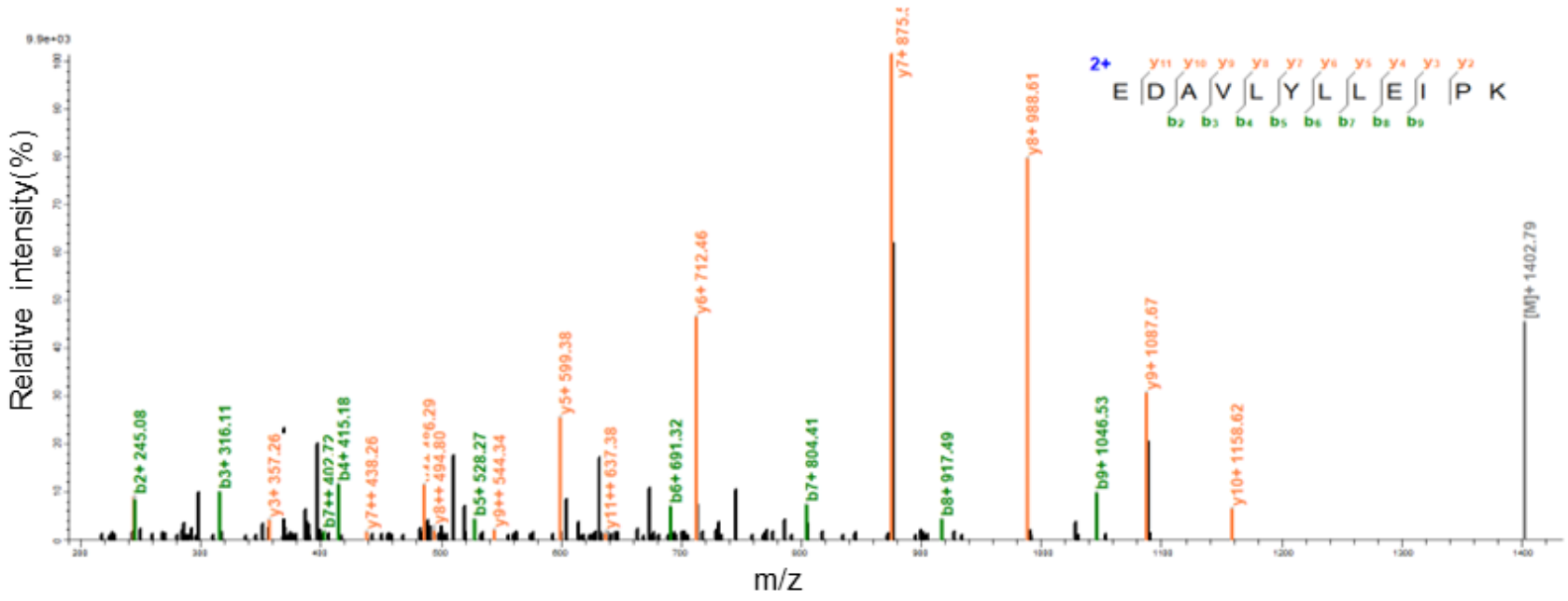

B

The secondary mass spectrums of PGAM5

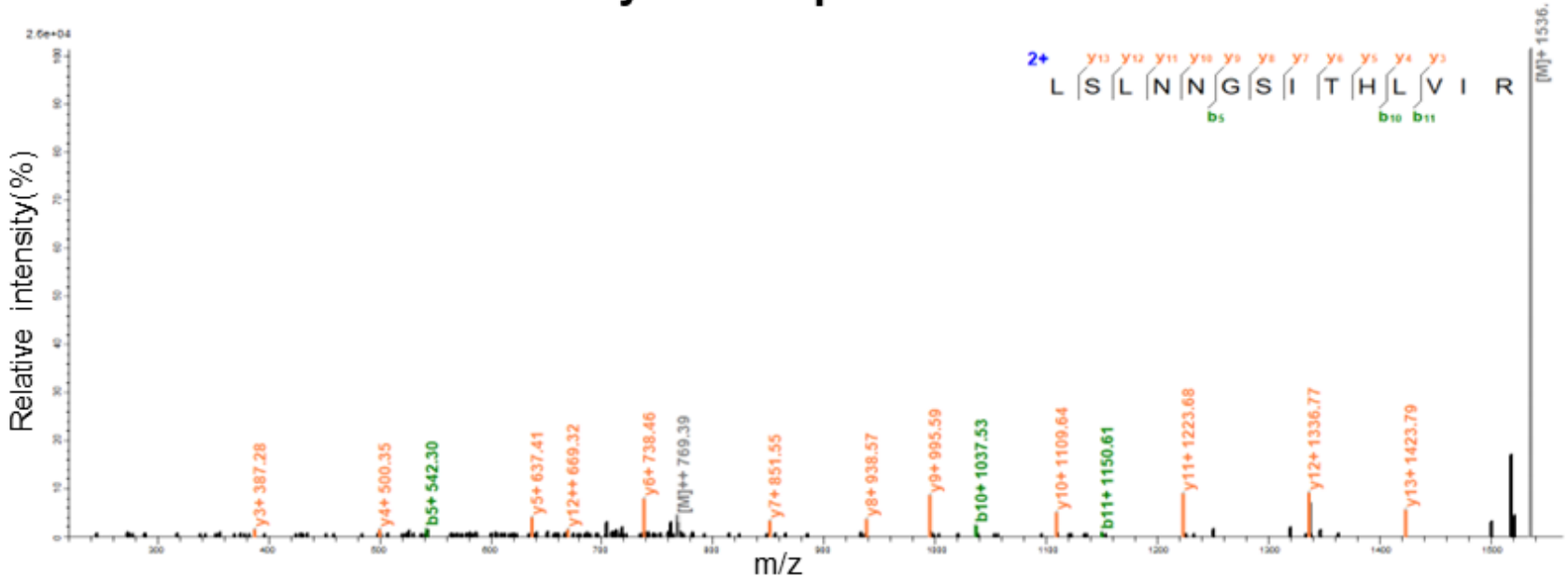

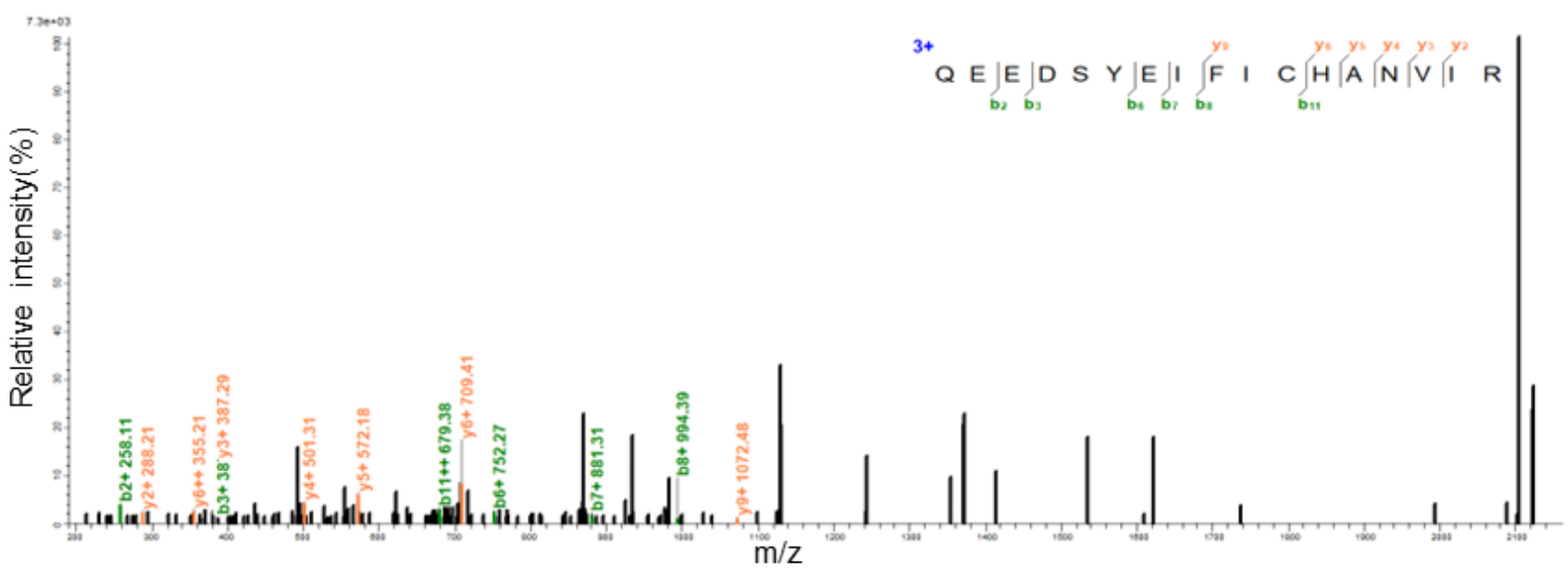

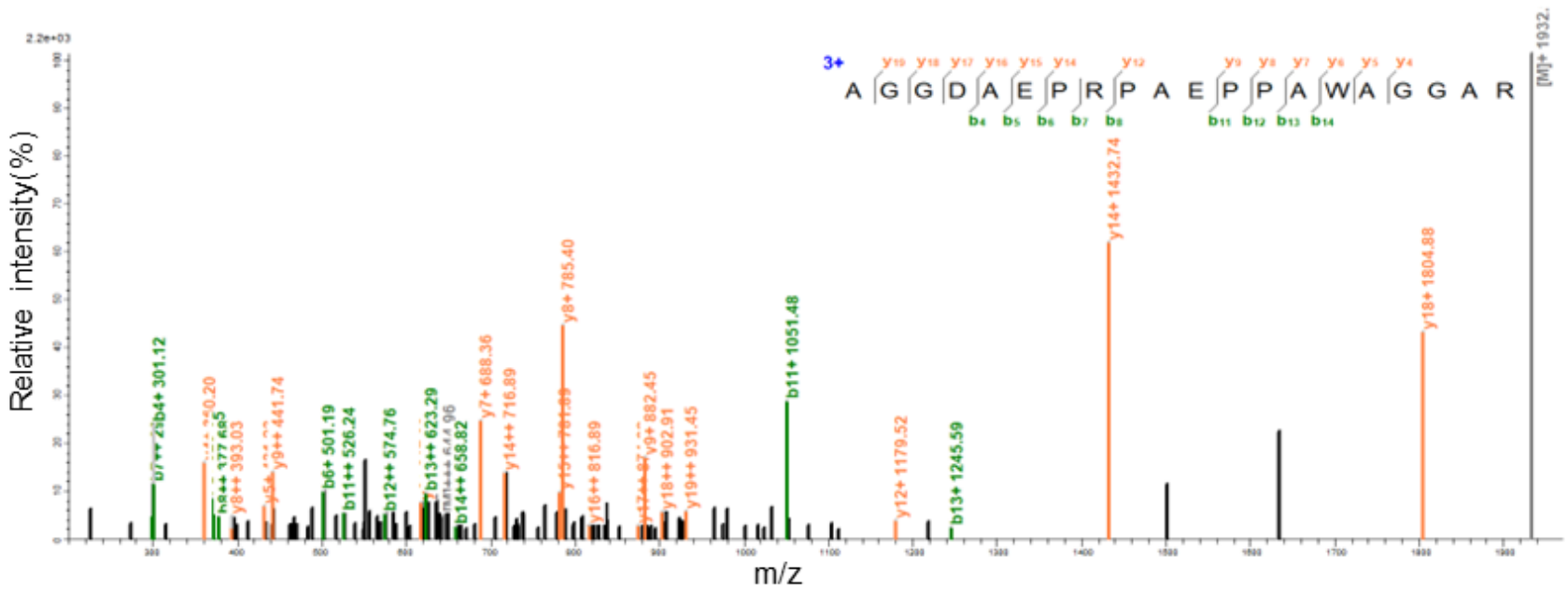

Fig. S4. The secondary mass spectrums of ZO-2 and PGAM5.

Figure S5

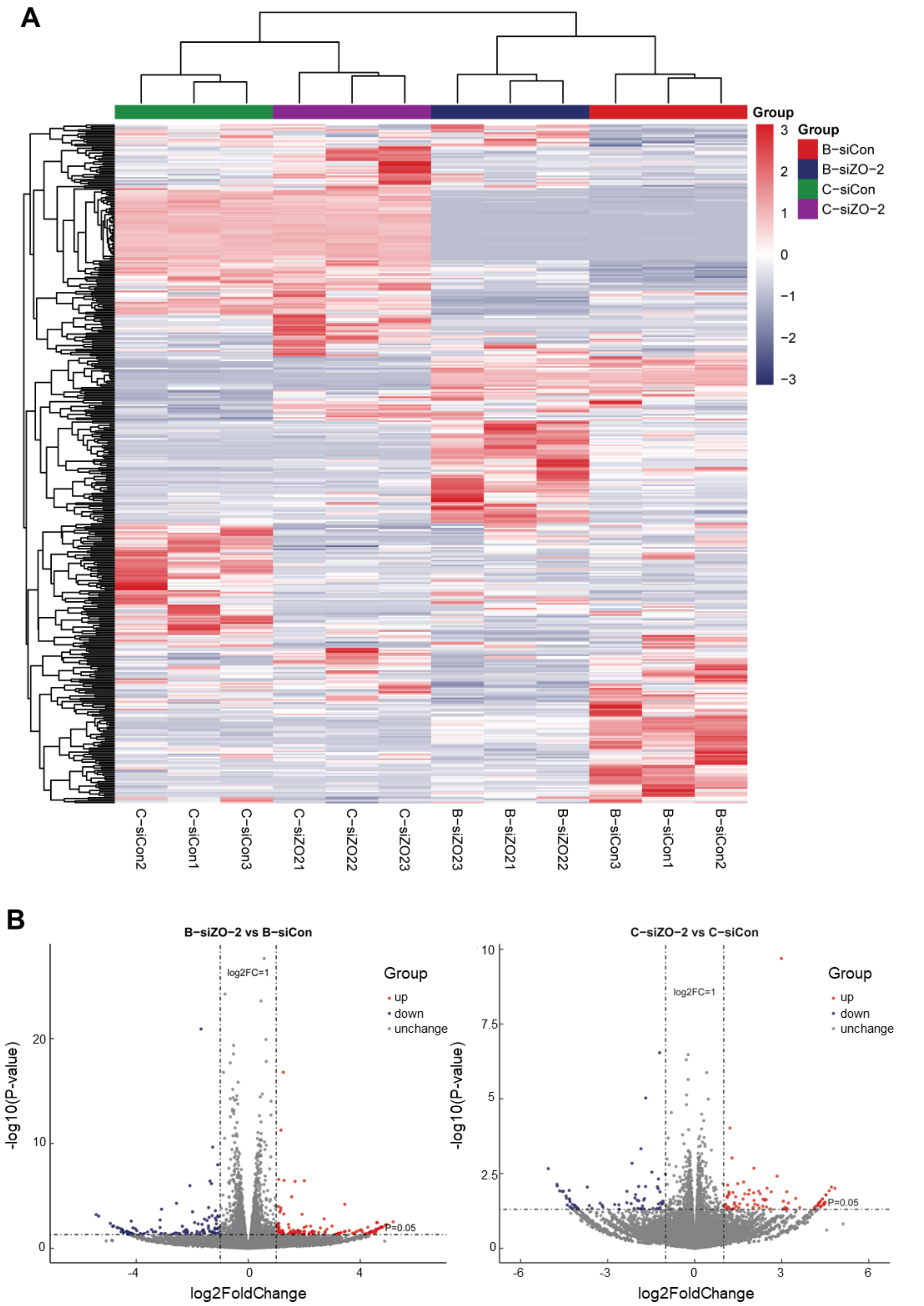

Fig. S5. Clustering analysis of differentially expressed genes after ZO-2 knockdown in PDAC cells.

(**A**) Clustering analysis after ZO-2 knockdown in BxPC-3 and CFPAC-1 cells (n=3). (**B**) Volcano plots of the differentially expressed genes after ZO-2 knockdown in BxPC-3 and CFPAC-1 cells (n=3).

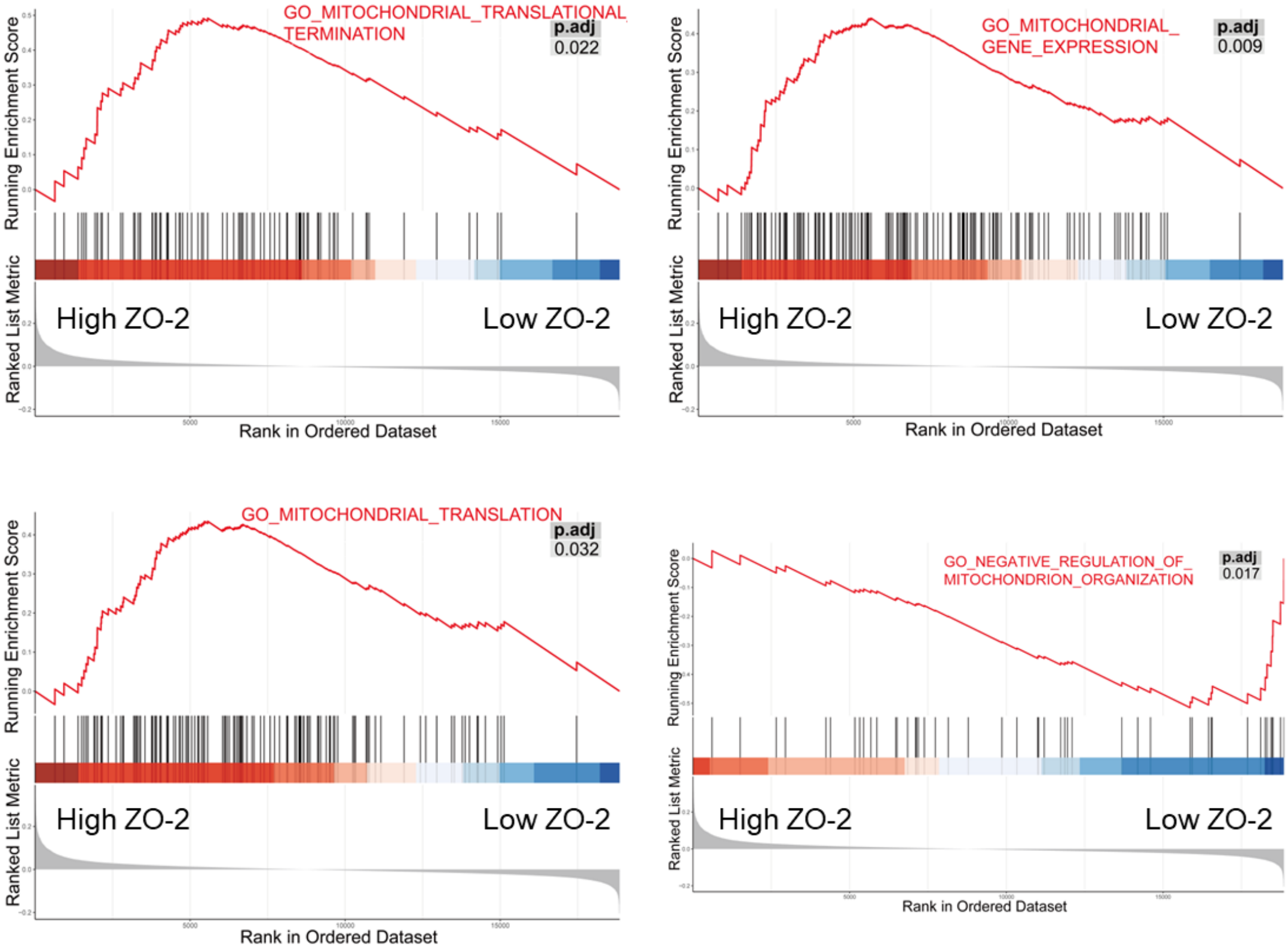

Fig. S6. ZO-2 was associated with mitochondrial function.

Gene set enrichment analysis (GSEA) of the public PDAC dataset (GSE62452) according to the ZO-2 expression levels.

Figure S7

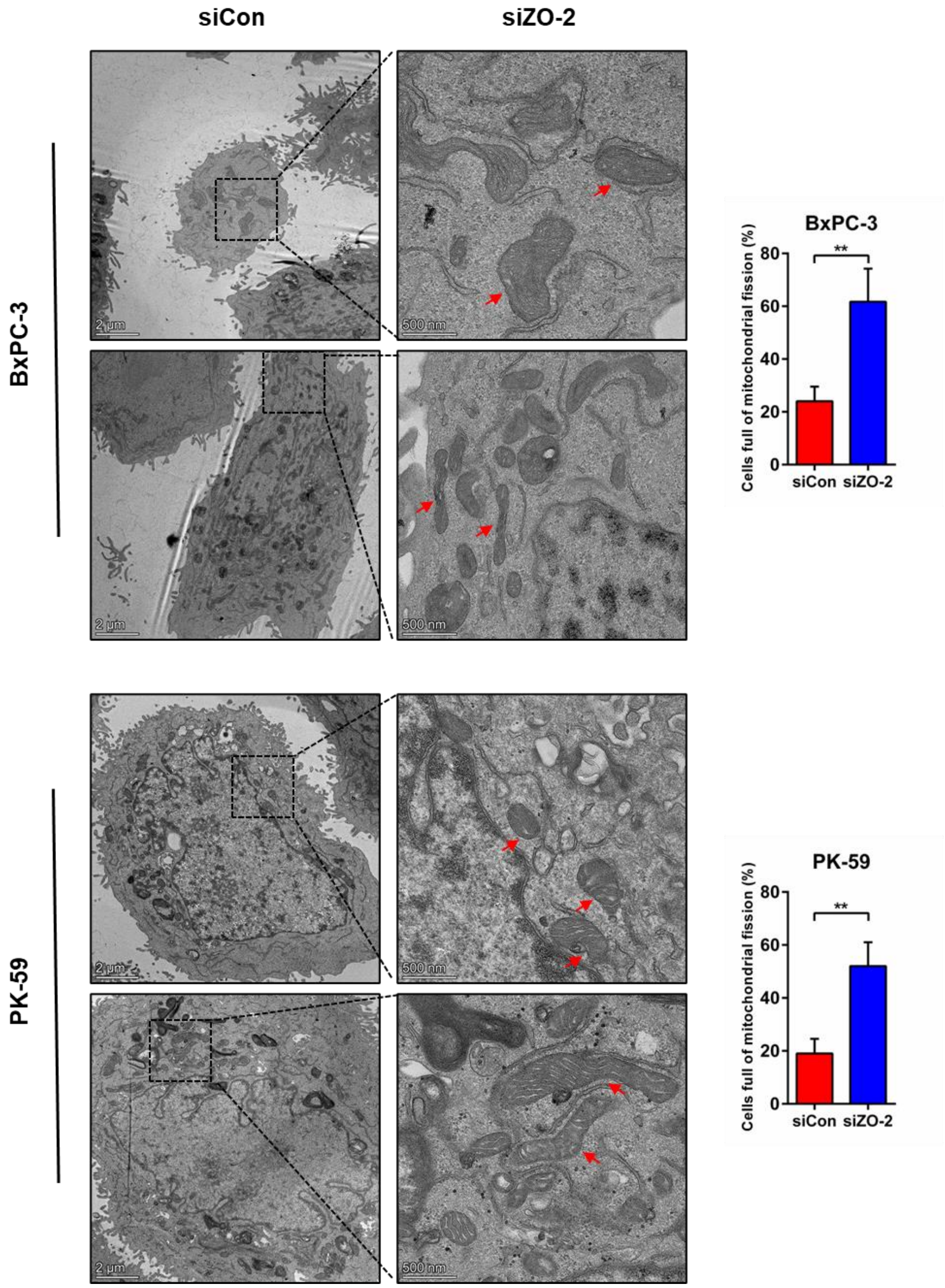

Fig. S7. The role of ZO-2 in mitochondrial morphology.

Mitochondrial morphology (red arrow) was observed with transmission electron microscopy (TEM) following ZO-2 knockdown in BxPC-3 and PK-59 cells. (n=3, scale bar, 2μm, 500nm).

Figure S8

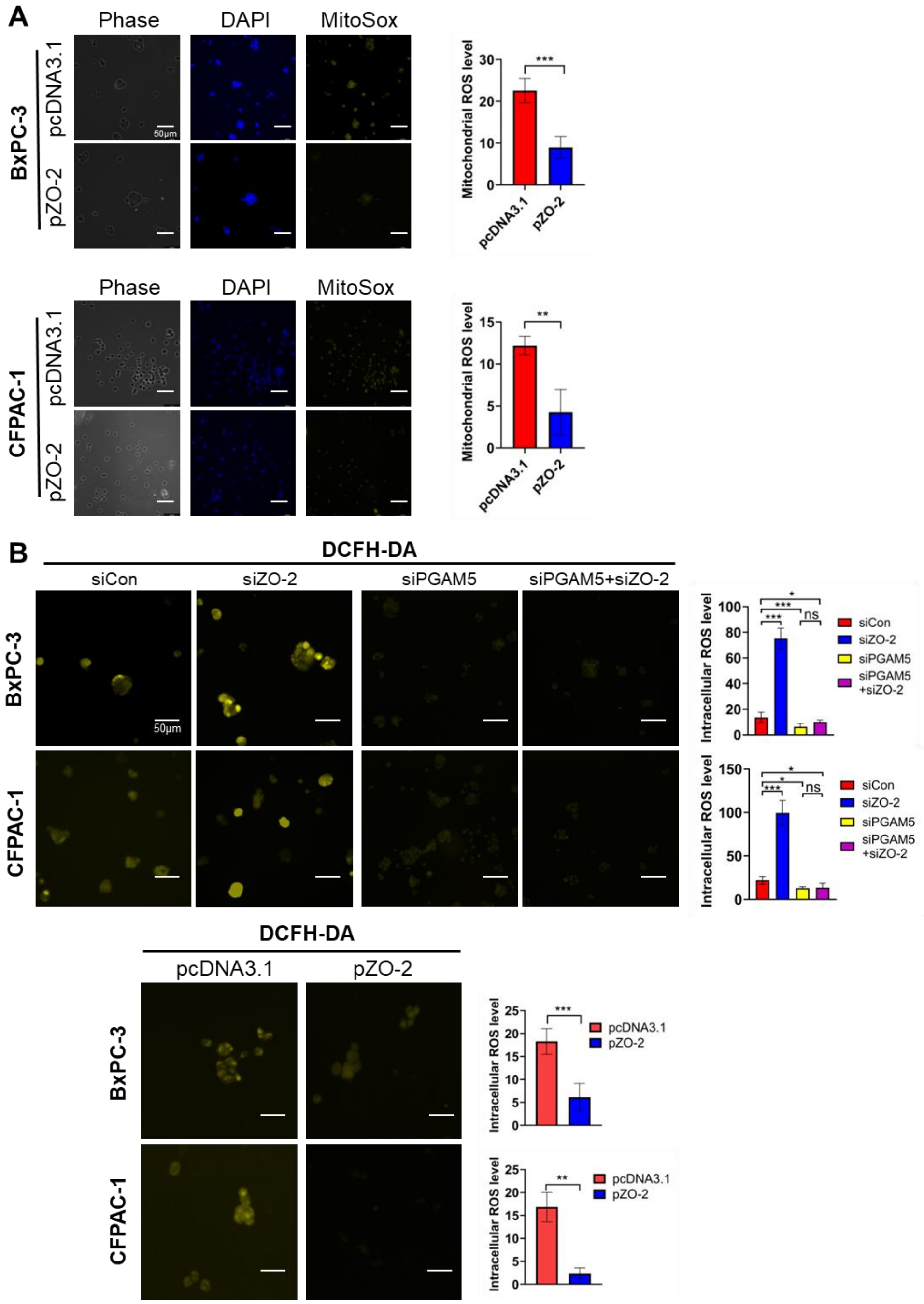

Fig. S8. The roles of ZO-2 and PGAM5 in mitochondrial and intracellular ROS levels of PDAC cells.

(**A**) Mitochondrial ROS levels were detected with the MitoSox fluorescent probe and the image J software in BxPC-3 and CFPAC-1 cells transfected with pcDNA3.1 or pZO-2 (20x magnification, n=3). (**B**) Intracellular ROS levels were detected with the DCFH-DA fluorescent probe and the image J software in BxPC-3 and CFPAC-1 cells transfected with siCon, siZO-2, pcDNA3.1, pZO-2, siPGAM5 or siPGAM5+siZO-2 (20x magnification, n=3).

Figure S9

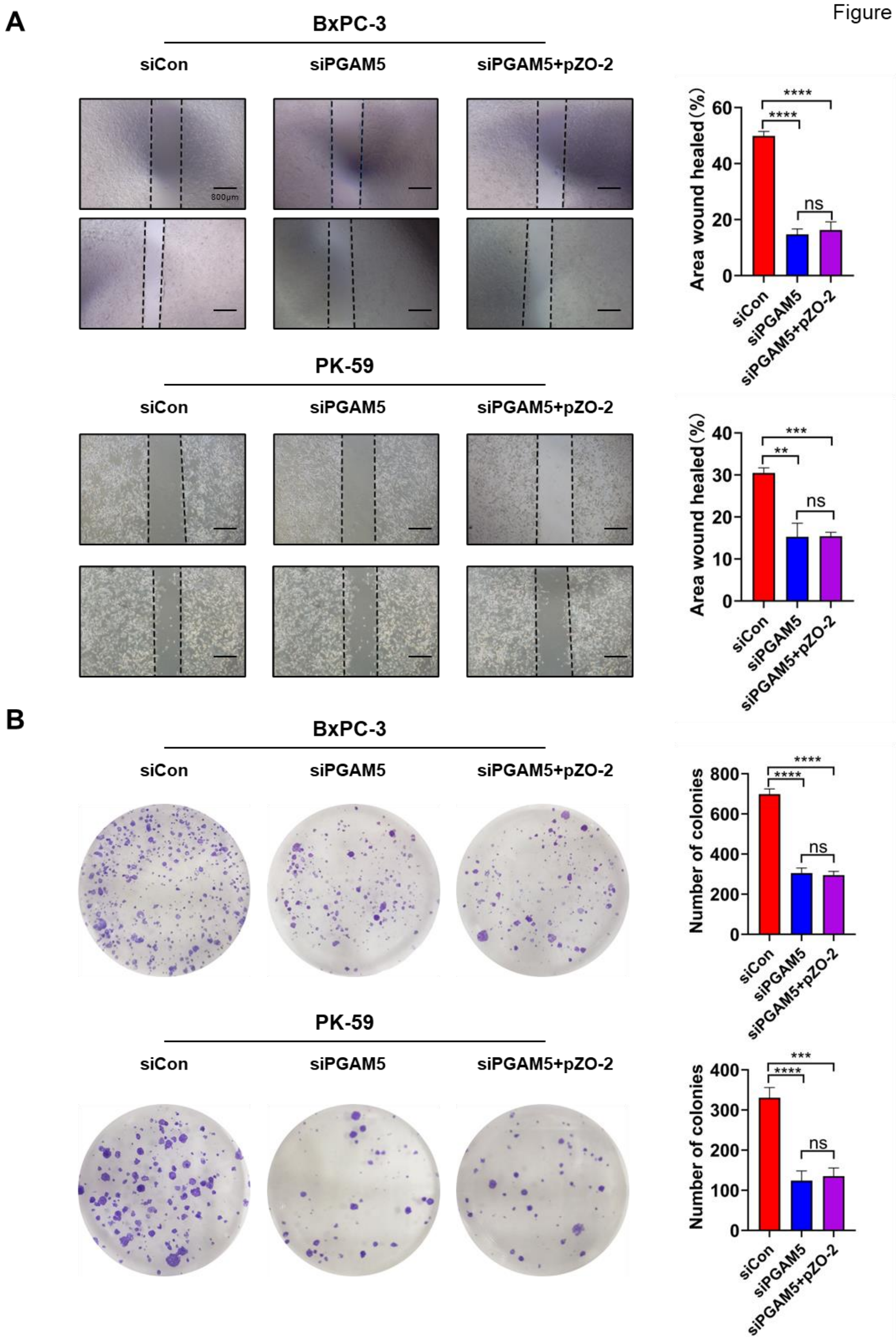

Fig. S9. ZO-2 inhibited human pancreatic cancer malignancy through PGAM5. (**A**) The wound areas of BxPC-3 and PK-59 cells transfected with siCon, siPGAM5 or pZO-2 were measured with the image J software after scraping cells with 200 μL tips (5x magnification, n=3). (**B**) Colonies formed by BxPC-3 and PK-59 cells transfected with siCon, siPGAM5 or pZO-2 were fixed with paraformaldehyde and stained with crystal violet, and colony numbers were determined with the image J software (n=3).

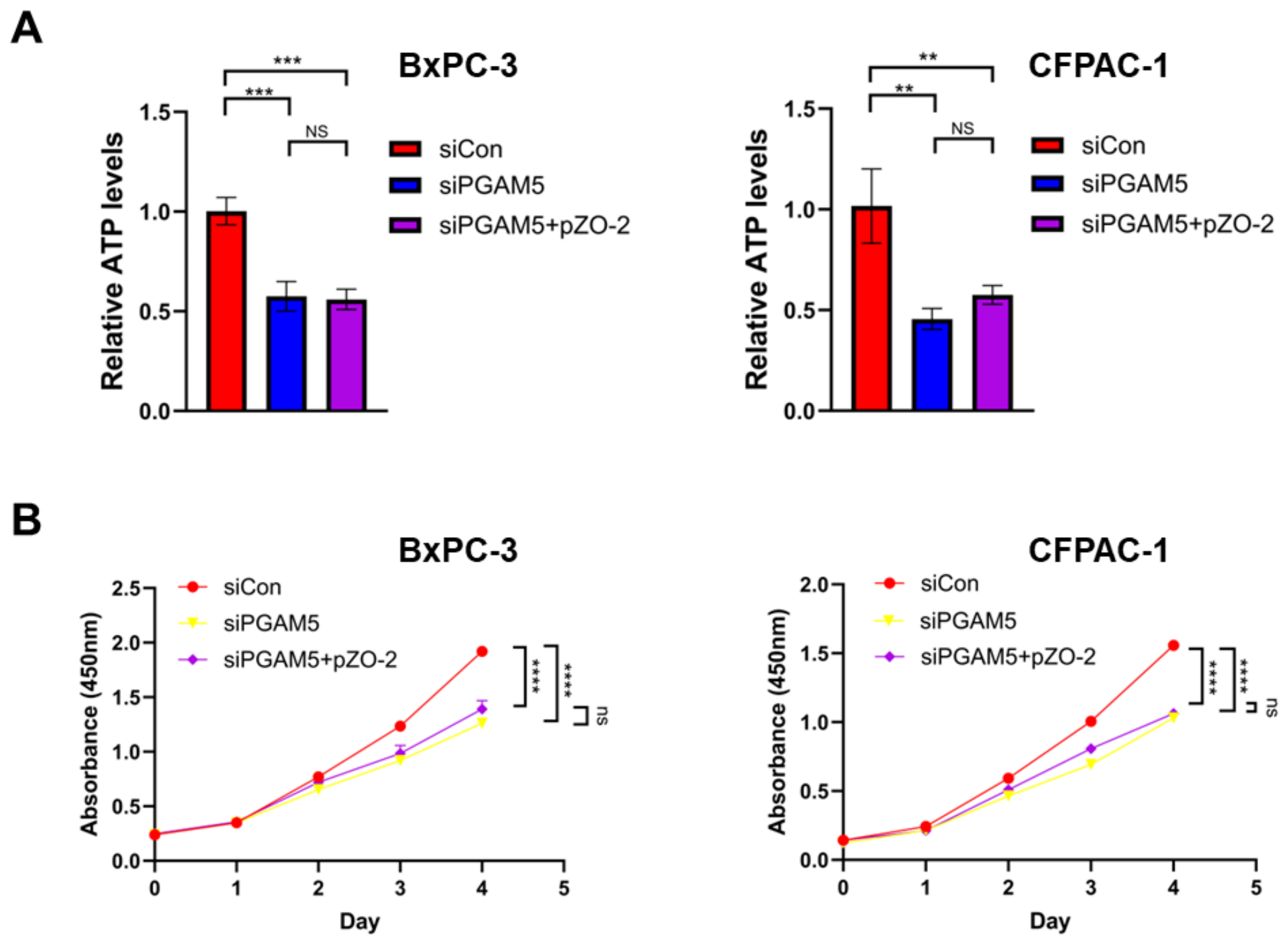

Fig. S10. ZO-2 impaired mitochondrial functions and cell viability through PGAM5.

(**A**) ATP levels were detected with luciferase and fluorescein in BxPC-3 and CFPAC-1 cells transfected with siCon, siPGAM5 or siPGAM5+pZO-2. (**B**) Viability of BxPC-3 and CFPAC-1 cells transfected with siCon, siPGAM5 or siPGAM5+pZO-2 was evaluated by the CCK-8 assay (n=3).

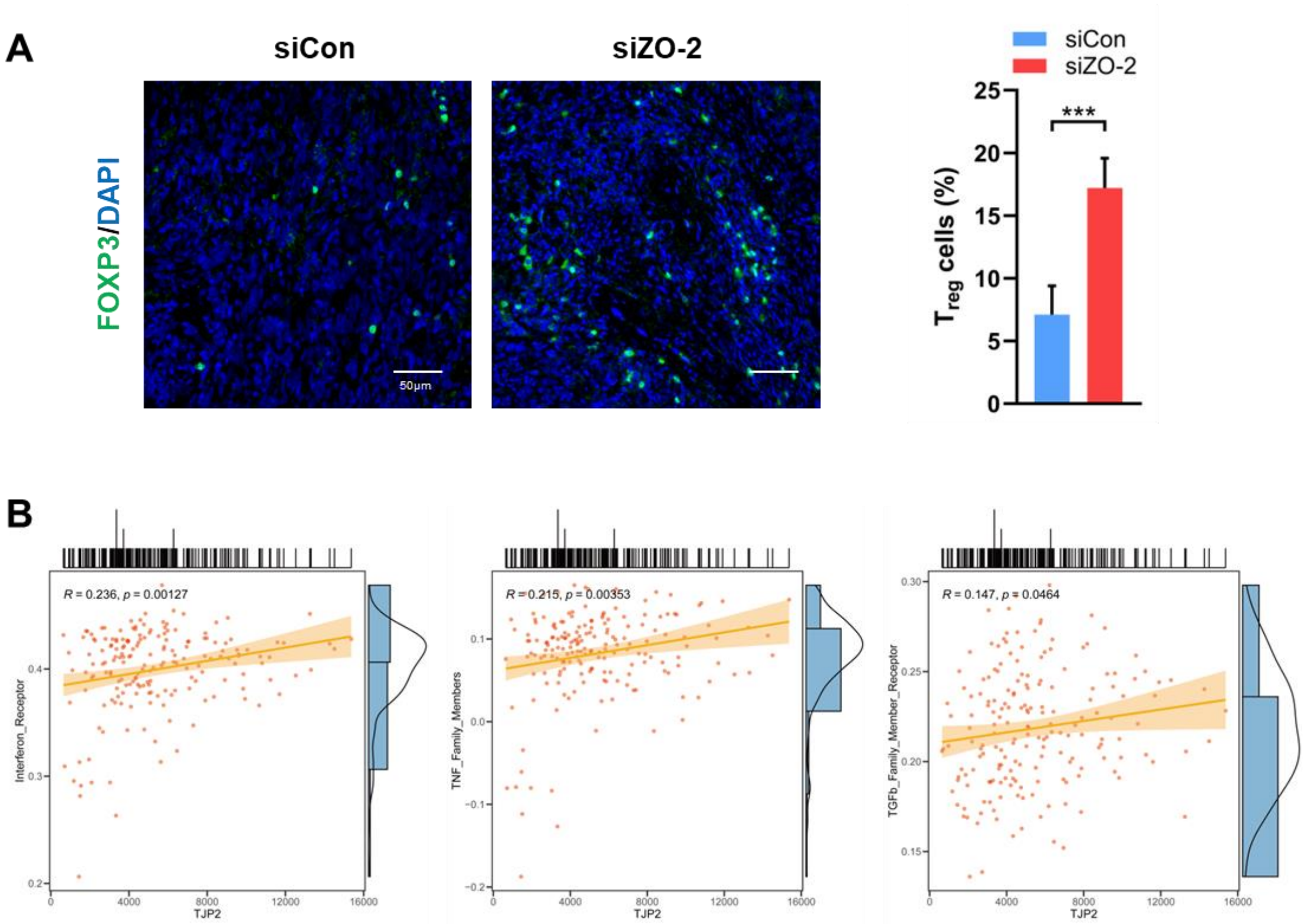

Fig. S11. ZO-2 was related with several immune signaling pathways.

(**A**) The fluorescence staining of FOXP3 in Panc02 tumors transfected with siCon or siZO-2 (40x magnification, n=3). (**B**) ZO-2 was related with several immune signaling pathways.

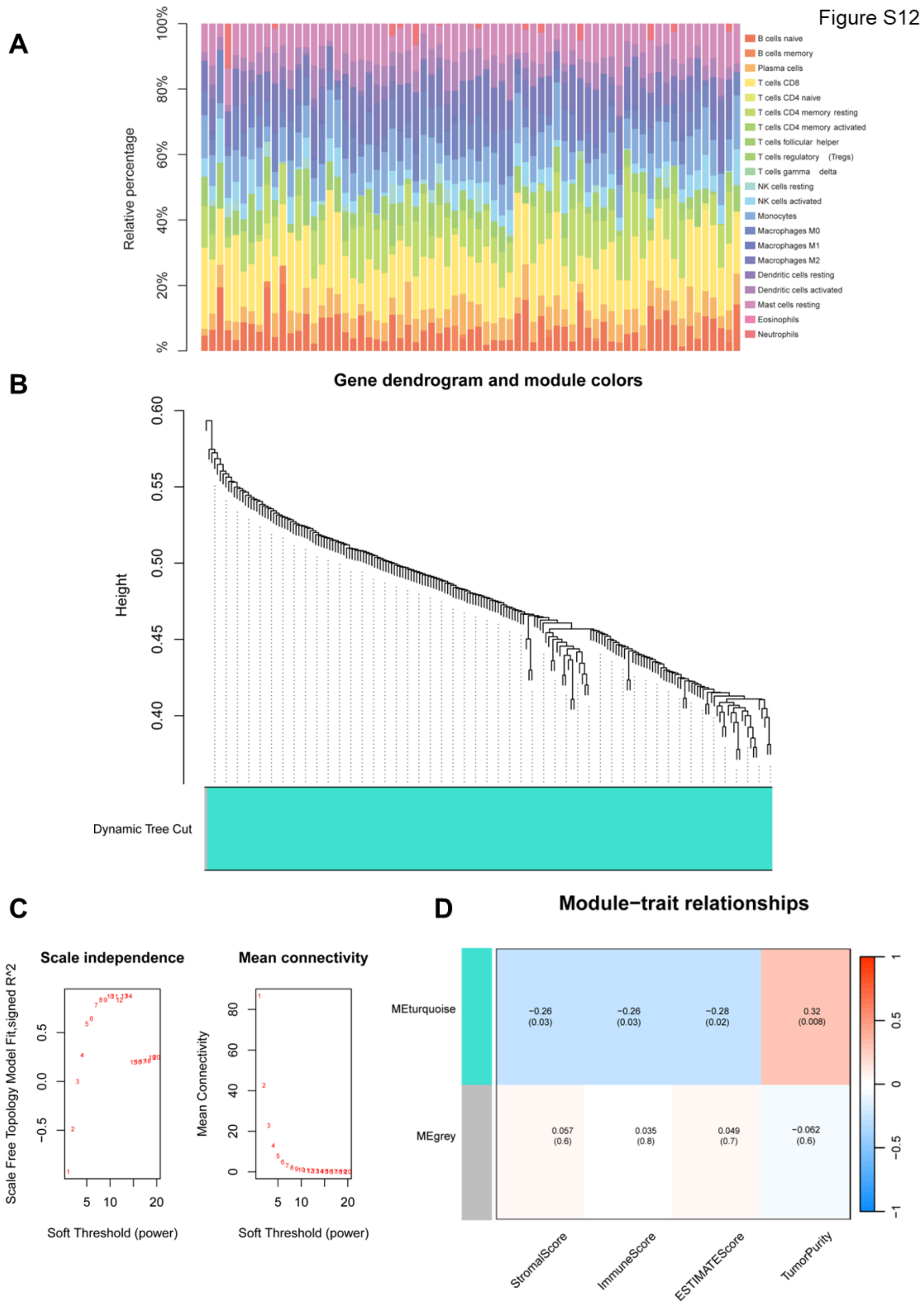

Fig. S12. Immune cell proportion and screening for modules and genes related to ZO-2 in the public PDAC dataset (GSE62452).

(**A**) Analysis of immune cell infiltration in PDAC tissues. (**B**) Clustering dendrogram of PDAC tissues. (**C**) Calculation of the scale-free fit index and the mean connectivity of various soft thresholding powers. (**D**) A correlation heatmap between module eigengenes and immune related parameters.

Figure S13

A

B

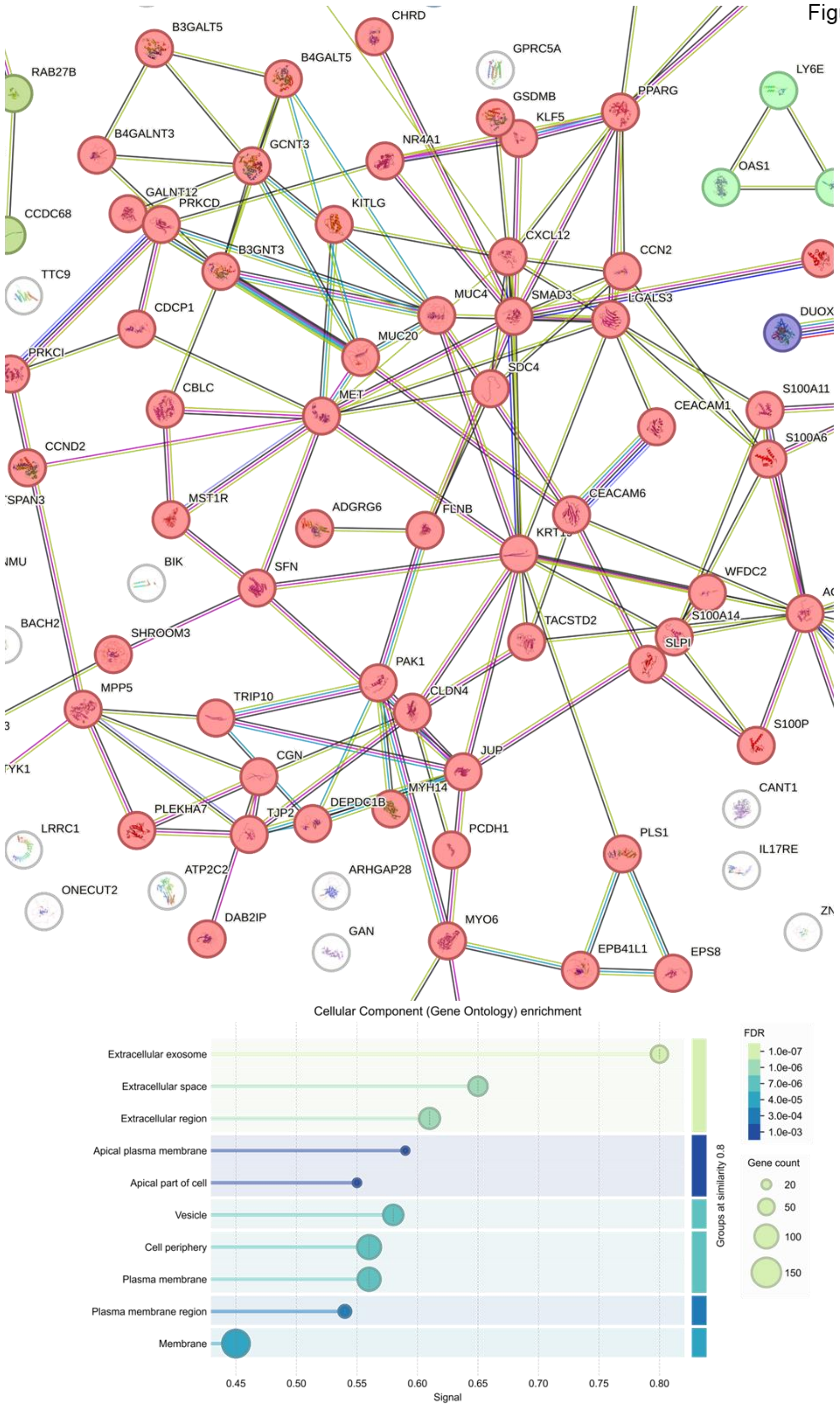

Fig. S13. PPI enrichment analysis of genes of the turquoise module.

(**A**) The PPI network of genes of the turquoise module. (**B**) The enrichment analysis of genes of the turquoise module.

Table S1. Mutational status of key driver genes of utilized PDAC cell lines

Cell lines / Genes	BxPC-3	CFPAC-1	PK-59	Panc-02
**KRAS**	-	G12V	G12D	G12D
**TP53**	Y220C	C242R	Loss	R248Q
**CDKN2A**	Loss	-	-	Y44*
**SMAD4**	Loss	Loss	-	R135*

-: No variants, Loss: Homozygous deletion, *: Stop codon
